# Genetic Characteristics of Korean Patients with Autosomal Dominant Polycystic Kidney Disease by Targeted Exome Sequencing

**DOI:** 10.1038/s41598-019-52474-1

**Published:** 2019-11-18

**Authors:** Hyunsuk Kim, Hayne Cho Park, Hyunjin Ryu, Hyunho Kim, Hyun-Seob Lee, Jongho Heo, Chung Lee, Nayoung K. D. Kim, Woong-Yang Park, Young-Hwan Hwang, Kyu Beck Lee, Kook-Hwan Oh, Yun kyu Oh, Curie Ahn

**Affiliations:** 1 Department of Internal Medicine, Chuncheon Sacred Heart Hospital, Chuncheon, Korea; 2 Department of Internal Medicine, Kangnam Sacred Heart Hospital, Seoul, Korea; 3 Department of Internal Medicine, Seoul National University Hospital, Seoul, Korea; 4 Center for Medical Innovation, Seoul National University Hospital, Seoul, Korea; 5 Genomic Core Facility, Transdisciplinary Research and Collaboration Division, Translational Research Institute, and Biomedical Research Institute, Seoul National University Hospital, Seoul, Korea; 6 National Assembly Futures Institute, Seoul, Korea; 7 Samsung Genome Institute, Samsung Medical Center, Seoul, Korea; 8 Department of Health Science and Technology, Samsung Advanced Institute for Health Sciences and Technology, Sungkyunkwan University, Seoul, Korea; 9 Department of Molecular Cell Biology, Sungkyunkwan University School of Medicine, Seoul, Korea; 10 Truewords Dialysis Clinic, Incheon, Korea; 11 Department of Internal Medicine, Kangbuk Samsung Hospital, Sungkyunkwan University School of Medicine, Seoul, Korea; 12 Department of Internal Medicine, Seoul National University Boramae Medical Center, Seoul, Korea

**Keywords:** Disease genetics, Polycystic kidney disease

## Abstract

Autosomal dominant polycystic kidney disease (ADPKD) is one of the main causes of end-stage renal disease (ESRD). Genetic information is of the utmost importance in understanding pathogenesis of ADPKD. Therefore, this study aimed to demonstrate the genetic characteristics of ADPKD and their effects on renal function in 749 Korean ADPKD subjects from 524 unrelated families. Genetic studies of *PKD1/2* were performed using targeted exome sequencing combined with Sanger sequencing in exon 1 of the *PKD1* gene and a multiple ligation probe assay. The mutation detection rate was 80.7% (423/524 families, 331 mutations) and 70.7% was novel. *PKD1* protein-truncating (*PKD1*-PT) genotype was associated with younger age at diagnosis, larger kidney volume, lower renal function compared to *PKD1* non-truncating and *PKD2* genotypes. The *PKD1* genotype showed earlier onset of ESRD compared to *PKD2* genotype (64.9 vs. 72.9 years old, P < 0.001). In frailty model controlled for age, gender, and familial clustering effect, *PKD2* genotype had 0.2 times lower risk for reaching ESRD than *PKD1*-PT genotype (p = 0.037). In conclusion, our results suggest that genotyping can contribute to selecting rapid progressors for new emerging therapeutic interventions among Koreans.

## Introduction

Autosomal dominant polycystic kidney disease (ADPKD) is a potentially lethal monogenic disorder characterized by numerous cysts in the kidneys and progressive renal failure^1^. Since the disease is caused by a mutation either in *PKD1* or *PKD2*, genetic information is of the utmost importance in understanding the pathogenesis of ADPKD.

The ADPKD mutation database (PKDB, http://pkdb.mayo.edu/) is the largest international repository for variants found in PKD genes. By July 2019, 1895 pathogenic mutations in the *PKD1* gene and 438 in the *PKD2* gene were registered in the PKDB. However, variants registered in the PKDB mainly came from the results of genetic studies in Western population. There have been only a few reports that presented genetic profiles on Asian ADPKD patients since analyzing *PKD1/2* mutations has been difficult in clinical practice. First, two-thirds of the 5′ region of *PKD1* are duplicated by gene conversion (GC) on chromosome 16 within six pseudogenes (*PKD1*P1-P6)^2^. Because these pseudogenes have a sequence that is 97.7% identical to the original *PKD1* gene sequence, cloning and sequencing *PKD1* has been difficult with direct sequencing. Moreover, high allelic heterogeneity of both *PKD1* and *PKD2* makes molecular diagnosis of ADPKD more challenging. Previously, indirect methods such as two-dimensional gene scanning, denaturing gradient gel electrophoresis, and single-strand conformation polymorphism analysis have been applied to find pathogenic mutations^3–6^. However, these indirect methods demonstrated false-positive results with a low detection rate compared to direct sequencing. Therefore, a long-range polymerase chain reaction (PCR) with subsequent nested PCR was developed to isolate the entire *PKD1* gene without interference from the six pseudogenes (*PKD1*P1-P6)^7^. However, direct Sanger sequencing is time-consuming and costly. Therefore, there has been an urgent need for faster and more efficient way to detect mutations in ADPKD.

Targeted exome sequencing (TES) using next-generation sequencing (NGS) is one of the most suitable methods to study monogenic disorders such as ADPKD. It has advantages over Sanger sequencing including that a large amount of data can be analyzed automatically in a short period of time and can be done cheaply. In addition, researchers can focus on a few genes and exons to discover mutation spots. Therefore, TES, rather than whole exome sequencing or whole genome sequencing, has been used more popularly as a genetic screening tool to detect mutations in ADPKD. Since TES became available in clinical practice for genetic diseases, the detection rate gradually improved^7^.

The importance of genotype on clinical outcome has been demonstrated from many previous studies. Cornec-Le Gall *et al*.^8^ reported the influence of *PKD1* mutation type on renal survival. HALT-PKD and CRISP Investigators showed the difference of renal survival in *PKD1* non-truncating (*PKD1*-NT) carriers according to their conservation and chemical change^9^. Previously, anecdotal report from Asian countries reported novel mutations in Asian ADPKD populations^10–12^. However, they were either case series or included small samples. No well-designed cohort studies have been conducted to evaluate genetic profiles and genotype-phenotype associations in Asian populations. The coHOrt for genotype-PhenotypE correlation in ADPKD (HOPE-PKD) is a large prospective, multicenter Korean ADPKD cohort constructed to investigate the natural course of the disease and genotype-phenotype associations. Demographic and clinical information of the subjects, as well as family trees, is collected, and specimens including DNA, serum, and urine sample are collected and stored from the subjects once a year^13^. To describe the genetic characteristics of ADPKD in Koreans, we performed a cohort-based genetic analysis using the HOPE-PKD cohort.

## Results

### Demographic characteristics of 749 subjects of the korean HOPE-PKD cohort

Among 749 subjects, 360 (48.1%) were males and 389 (51.9%) were females. The mean age was 46.4 ± 13.3 years (male, 44.6 ± 14.4; female, 48.0 ± 12.0; P value for gender difference, <0.001). The mean estimated glomerular filtration rate (eGFR) was 65.8 ± 38.9 mL/min/1.73 m^2^ (male, 65.6 ± 39.4; female, 66.0 ± 38.5; P value for gender difference, 0.896, see Supplementary Table S1).

### Utility of TES as a genetic screening method for pathogenic mutations

The validation set (n = 80) was developed to confirm the variants that was discovered by TES. Among 80 variants found by TES, 74 were *PKD1* variants and 6 were *PKD2*. Among 74 *PKD1* variants, 70 variants (94.6%) were detected in duplicated region (exon 1–33 in *PKD1* gene). For the duplicated part of *PKD1*, long-range PCR followed by nested-PCR was performed using *PKD1*-specific primers, as previously described^11^. Among them, 79 variants were confirmed by Sanger sequencing (98.8%). The results are summarized in the Supplementary Table S2. The unconfirmed variant was a missense variant c.C2081T (p.Pro694Leu) in exon 10 of *PKD1*. Although we did not perform long-range PCR/Sanger sequencing for whole *PKD1/PKD2* regions, the validation study suggests TES is efficient in variant detection even in the duplicated region.

Next, we applied TES to the whole HOPE-PKD cohort to evaluate the efficacy of TES for genetic analysis. In order to overcome pseudogene issue and low coverage, we used several approaches: (1) the bait was tiled 3 times (see Supplementary Table S3), (2) only selected reads with a high mapping quality (>30) were used, 3) only variants with low allele frequency <1% were used, and 4) variants with either low quality reads (<20) or low genotype quality (<30) were excluded (see Supplementary Fig. S1). As the results, the mean coverage of *PKD1* was 1523x and that of PKD2 was 1422x (see Supplementary Table S4). The TES approach alone discovered 359 variants from 471 out of 524 families. Among them, definitively pathogenic (DP), highly likely pathogenic (HLP), or likely pathogenic (LP) variants were categorized as mutations. Thus, mutations were found in 409 families. Therefore, mutation detection rate for TES alone was 78.1% (409/524).

### *PKD1* Exon 1-specific sanger sequencing, multiple ligation probe assay, and familial segregation analysis to improve mutation detection rate

In 62 families with likely neutral (LN) or indeterminate (I) variants and 53 families with no variants (NV), subsequent *PKD1* exon 1 Sanger sequencing and/or multiple ligation probe assay (MLPA) were carried out. In LN or I families, six mutations were additionally detected through *PKD1* exon 1 Sanger sequencing, and three mutations were detected through MLPA. In NV families, one and four mutations were found through *PKD1* exon 1 Sanger sequencing and MLPA, respectively. Among 129 families with novel LP variants found by TES, 88 families underwent additional familial segregation analysis. Forty families were determined to have HLP mutations, and 2 families were proven to have LN mutations. Thus, the mutation detection rate was 80.7% (423/524 families) by TES, *PKD1* exon 1 Sanger sequencing, MLPA, and familial segregation analysis (see Supplementary Fig. S2).

### Baseline clinical characteristics according to PKD genotypes and summary of identified mutations

**Figure 1:**
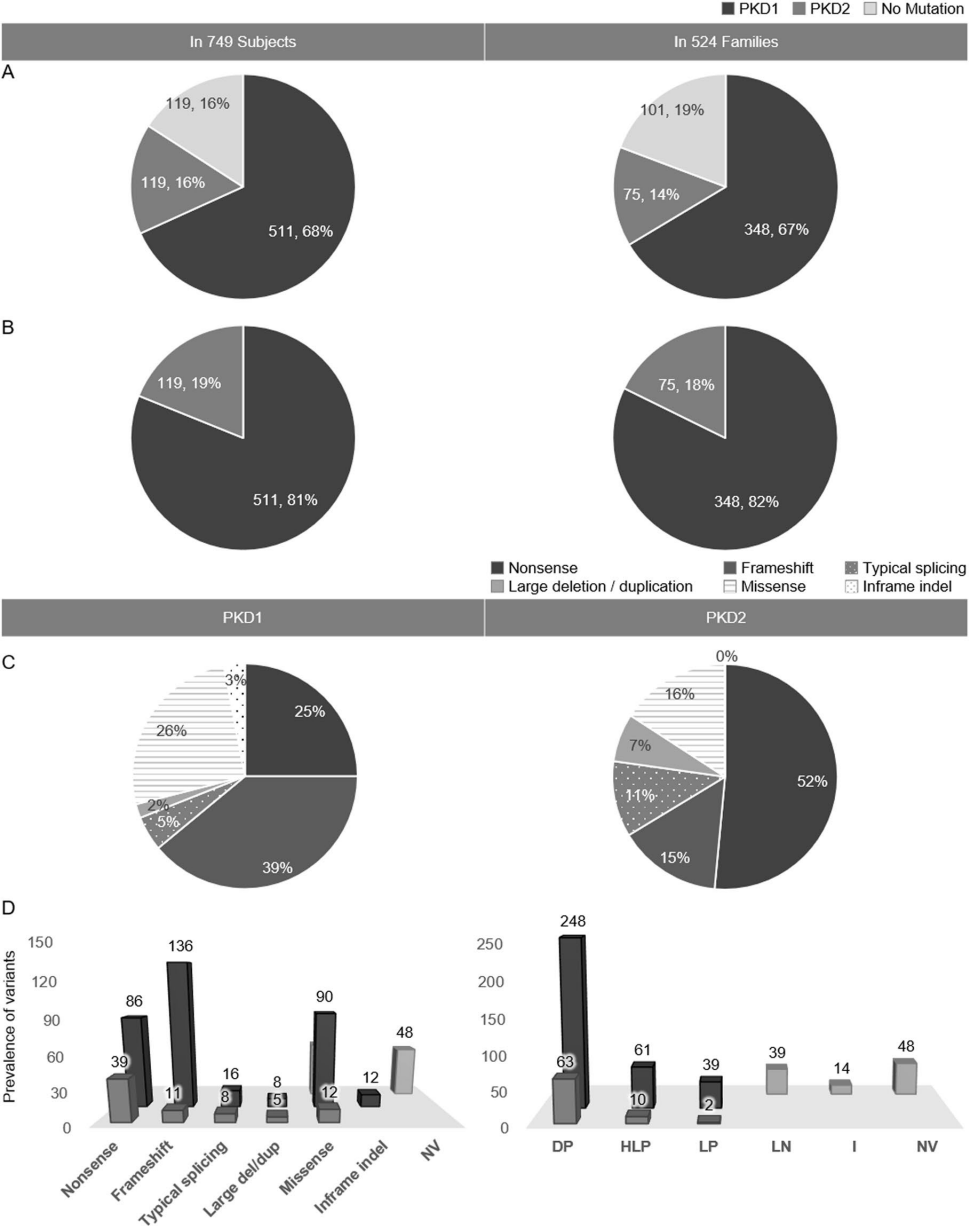
Summary of identified mutations. (**A**) Prevalence of *PKD1* vs. *PKD2* mutations and cases of no mutation among 749 subjects (524 families); (**B**) Prevalence of *PKD1* vs. *PKD2* mutations among 630 subjects (423 families without NM families [n = 101]); (**C**) Frequency of each class in 423 families according to *PKD1/2* status; D. Frequency of each mutation class and grade in 524 families. Of the 749 subjects, 68% were classified as *PKD1*, 16% as *PKD2*, and 16% as no mutation (NM). Excluding NM subjects, 81% of the 630 subjects and 82% of the 423 families had *PKD1* mutations. The mutation classes in the 423 families that were obtained by excluding the 101 NM families from the original 524 families are shown in (**C**). In *PKD1*, frameshift mutations were the most common (39%), and in *PKD2*, nonsense mutations were the most common (52%). The prevalence of classes of variants (number of families that shared the same variants) among the 524 families was shown in (**D**). *Abbreviations*. DP, definitively pathogenic; HLP, highly likely pathogenic; LP, likely pathogenic; LN, likely neutral; I, indeterminate; NV, no variant.

A total of 364 variants were found in 476 of the 524 families, and of these, 331 mutations were detected among 423 families in the HOPE-PKD cohort (Fig. 1). Among the 749 subjects, 68% had *PKD1* variants, 16% had *PKD2* variants, and 16% were classified as having no mutations (NM). Excluding NM, 81% of the 749 subjects and 82% of the 524 families had *PKD1* mutations. The frameshift mutation (39%) was the most common mutation class in the *PKD1* genotype, while nonsense mutations (52%) were most common in the *PKD2* genotype. The prevalence of classes of variants (number of families that shared the same variants) among the 524 families was as follows: *PKD1*: nonsense, 86 (19); frameshift 136 (24), missense, 90 (17); *PKD2*: nonsense, 39 (20); frameshift, 11 (0); missense, 12 (4). The prevalence of the grades of the variants (number of families that shared the same variants) in the 524 families was as follows: *PKD1*: DP, 248 (46); HLP, 61 (20); and LP (0), 39; *PKD2*, DP, 63 (22); HLP, 10 (4); and LP, 2 (0) (Fig. 1 and Table 1).

**Table 1:** Frequency of each class and grade in 524 families.

A. Class	Nonsense	Frameshift	Typical splicing	Large del/dup	Missense	In-frame indel	NV
■ PKD1	86 (19)	136 (24)	16 (3)	8 (0)	90 (17)	12 (3)	
PKD2	39 (20)	11 (0)	8 (1)	5 (1)	12 (4)	0 (0)	
NM					53 (20)		48
Total	125 (39)	147 (24)	24 (4)	13 (1)	155 (41)	12 (3)	524 (112)
**B. Grade**	**DP**	**HLP**	**LP**	**LN**	**I**	**NV**	
■ PKD1	284 (46)	61 (20)	39 (0)				
PKD2	63 (22)	10 (4)	2 (0)				
NM				39 (19)	14 (1)	48	
Total	311 (68)	71 (24)	41 (0)	39 (19)	14 (1)	524 (112)	

The number in () means the number of families that shared the same variants

*Abbreviation*. DP, definitively pathogenic; HLP, highly likely pathogenic; LP, likely pathogenic; LN, likely neutral; I, indeterminate; NV, no variant

### Comparison of mutation frequency between HOPE-PKD and other cohorts

We compared the frequency of mutation between our HOPE-PKD cohort and other previously reported cohort (HALT population and Genkyst cohort). The results are described in Supplementary Table S5. Our cohort showed similar proportion of *PKD1* vs. *PKD2* mutations revealing 82% of *PKD1* genotype and 18% of *PKD2* genotype. Compared to Caucasian, Korean ADPKD cohort showed higher proportion of *PKD1*-PT and lower proportion of *PKD1*-NT.

Among the 331 mutations, 97 were registered in either the PKDB or Toronto Genetic Epidemiology Study of PKD^14^. Therefore 70.7% (234/331) of the mutations were novel (see Supplementary Table S6). Mutations were found more frequently in the C-type lectin and G protein-coupled receptor proteolytic site (GPS) domains of polycystin-1 in the Korean HOPE-PKD cohort than in those previously reported from PKDB. Likewise, mutations were detected more frequently in the linker domain of polycystin-2 in our patients compared to those in the PKDB. The standardized residual of the Chi-square test (cut-off of significance, 2.58) was 2.8 in the C-type lectin of polycystin-1 and the linker domain of polycystin-2, and 2.6 in the GPS domain of polycystin-1 (see Supplementary Fig. S3).

### Baseline clinical characteristics according to PKD genotypes

For genotype-phenotype analysis, *PKD1* or *PKD2* mutations were further classified into four PKD genotypes according to the previously described manner^14^; *PKD1* protein-truncating (*PKD1*-PT), *PKD1* small (fewer than five amino acids) in-frameshift indels (*PKD1*-ID), *PKD1* non-truncating (*PKD1*-NT), and *PKD2* genotypes. A total of 630 patients in 423 families after excluding NM were re-classified into four genotype groups: *PKD1*-PT, 371 (59%); *PKD1*-ID, 21 (3%); *PKD1*-NT, 119 (19%); *PKD2*, 119 (19%). *PKD1*-PT genotype was associated with younger age at the first visit (40.2 ± 12.9), earlier onset of hypertension (37.3 ± 10.1), younger ESRD age (52.5 ± 9.5), larger height-adjusted total kidney volume (htTKV) (898 [476, 1546], mL/m), and lower eGFR (63.5 ± 42.2, mL/min/1.73 m^2^) compared to other genotypes. The median follow-up duration was 77.3 months (Table 2).

**Table 2:** Baseline Clinical Characteristics According to PKD Genotypes.

Variables		Total	PKD1-PT	PKD1-ID	PKD1-NT	PKD 2	*P value*
N (%)		630	371 (59.0)	21 (3.0)	119 (19.0)	119 (19.0)	
Male, n (%)		304 (48.3)	175 (47.2)	12 (57.1)	68 (57.1)	49 (41.2)	*0.975*
Age, [mean ± SD]		45.6 ± 12.9	44.2 ± 12.9	45.0 ± 10.6	43.3 ± 12.6	48.9 ± 13.0	*0.001*
1st visit age, 569, [mean ± SD]		41.9 ± 12.9	40.2 ± 12.9	39.8 ± 10.0	43.3 ± 12.6	45.9 ± 13.0	<*0.001*
Hypertension, 590, n (%)		451 (71.6)	266 (71.7)	16 (80.0)	88 (77.2)	81 (75.7)	*0.969*
Hypertension Dx age, 316, [mean ± SD]		38.8 ± 10.0	37.3 ± 10.1	38.6 ± 7.4	39.0 ± 8.9	43.4 ± 10.5	<*0.001*
ESRD		122 (19.4)	84 (22.6)	4 (19.0)	22 (18.5)	12 (10.1)	*0.004*
Median age of ESRD		53.6	63.5	72.7	68.8	72.9	<*0.001*
Death		23 (3.7)	13 (3.5)	1 (4.8)	5 (4.2)	4 (3.4)	*0.908*
Death age, 23, [mean ± SD]		62.8 ± 13.8	61.4 ± 16.2	63.4	63.9 ± 9.1	65.7 ± 14.6	*0.745*
htTKV, median [IQR]		781 [432, 1356]	898 [476, 1546]	901 [544, 1271]	609 [397, 1179]	629 [373, 1160]	<*0.001*
eGFR by CKD-EPI, 620, [mean ± SD]		65.9 ± 40.0	63.5 ± 42.2	64.7 ± 38.3	66.6 ± 37.8	73.1 ± 33.8	*0.052*
*Grade, [n(%)]	1A	44 (7.0)	18 (4.9)	1 (4.8)	14 (11.8)	11 (9.2)	*<0.001*
	1B	158 (25.1)	70 (18.9)	2 (9.5)	35 (29.4)	51 (42.9)	
	1C	206 (32.7)	127 (34.2)	11 (52.4)	36 (30.3)	32 (26.9)	
	1D	153 (24.3)	99 (26.7)	7 (33.3)	25 (21)	22 (18.5)	
	1E	69 (11.0)	57 (15.4)	0 (0)	9 (7.6)	3 (2.5)	

P for trend by the Jonckheere-Terpstra test.

The eGFR of subjects with dialysis or kidney transplantation was regarded as 15 mL/min/1.73 m^2^.

*Abbreviations*. PT, protein-truncating; ID, insertion/deletion; NT, non-truncating, SD, standard deviation; Dx, diagnosis, ESRD, end-stage renal disease; eGFR, estimated glomerular flow rate; CKD-EPI, Chronic Kidney Disease Epidemiology Collaboration

As the Mayo imaging classification changed from 1A to 1E, the proportion of *PKD1*-PT genotype increased. Conversely, as the genotype changed from *PKD2* to *PKD1*-PT, the proportion of Mayo imaging classification 1C–E increased (P < 0.001 by linear by linear test, see Supplementary Fig. S4). During 77.3 months of follow-up, the intraclass correlation of individuals was 0.84 [0.82, 0.86] (See Supplementary Fig. S4). Therefore, the Mayo imaging classification of each subject was mostly retained over time.

When we applied the PROPKD score to our cohort, our cohort showed similar prognosis according to the PROPKD risk categories. As Supplementary Fig. S5 shows, renal survival was significantly different among three PROPKD prognostic categories, as follows: low risk (0–3 points), intermediate risk (4–6 points), and high risk (7–9 points). The median age of ESRD was 79 for low risk, 60 for intermediate risk, and 55 for high risk patients.

### HtTKV and eGFR according to PKD genotypes

**Figure 2:**
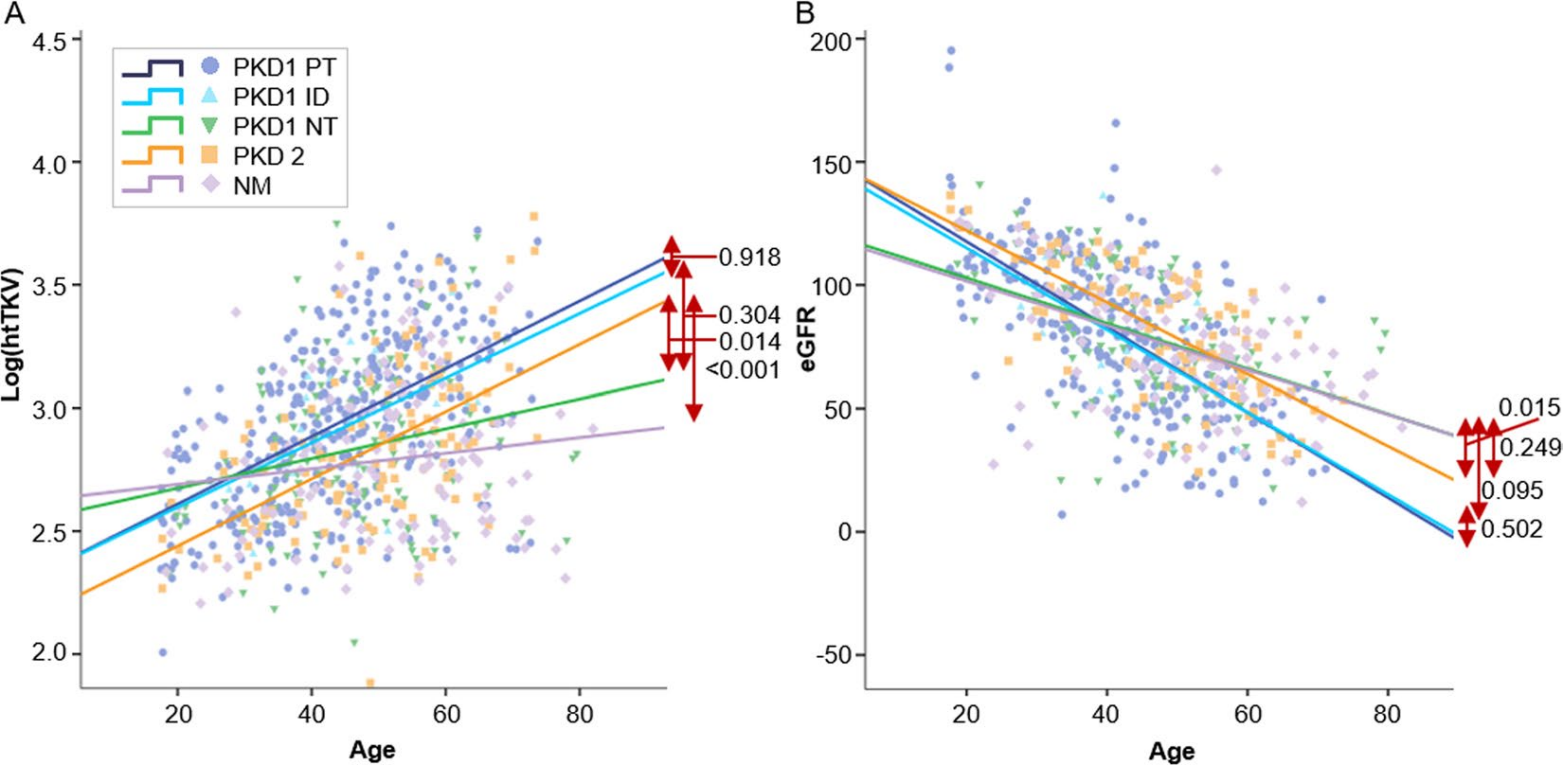
Cross-sectional analysis between age and log(htTKV) or eGFR (**A**). Scatter plot and trend line of log (htTKV) according to genotype. (**B**) Scatter plot and trend line of eGFR according to genotype. *PKD1*-PT genotype had a larger htTKV or lower eGFR than the *PKD1*-NT or *PKD2* genotypes. log(htTKV) increased rapidly with age in the *PKD1*-PT and *PKD2* genotypes, but it did so less intensely in the *PKD1*-NT genotype and those with NM. The *PKD1*-PT genotype also showed a steeper slope for eGFR than the other genotypes except *PKD1*-ID genotype. *Abbreviations*. htTKV, height-adjusted total kidney volume; eGFR, estimated glomerular filtration rate; *PKD1*-PT, *PKD1* protein-truncating; *PKD1*-NT, *PKD1* non-truncating; *PKD1*-ID, *PKD1* small in-frameshift indels.

When log(htTKV) was plotted according to age, the *PKD1*-PT genotype showed a similar slope to *PKD1*-ID and *PKD2* genotypes (Fig. 2). However, the *PKD1*-PT and *PKD1*-ID genotypes showed larger htTKV values compared to *PKD2* genotype (Table 3). Overall, the *PKD1*-PT and *PKD2* genotypes showed a steeper slope than the *PKD1*-NT genotype and NM group (Table 3). In other words, the log(htTKV) in the *PKD1*-PT and *PKD2* genotypes increased rapidly as age increased, but the log(htTKV) of the *PKD1*-NT genotype and NM group increased less intensely. The *PKD1*-PT genotype also showed a steeper slope of eGFR than the other genotype groups, except the *PKD1*-ID genotype (Fig. 2 and Table 3).

**Table 3:** A. Multilevel multivariate linear regression of log(htTKV) or eGFR according to genotype B. Multivariate linear regression of log(htTKV) or eGFR for comparing each slope according to genotype.

A.	Log(htTKV)			eGFR		
*Variable	Coefficient [95% CI]		*P value*	Coefficient [95% CI]		*P value*
Age	0.012 [0.010, 0.013]		<*0.001*	−1.840 [−2.012, −1.668]		<*0.001*
Male	0.102 [0.062, 0.143]		<*0.001*	−6.535 [−11.030, −2.041]		<*0.001*
PKD1 PT	reference			reference		
PKD1 ID	−0.033 [−0.186, 0.120]		*0.670*	2.668 [−112.914, 17.250]		*0.720*
PKD1 NT	−0.153 [−0.220, −0.085]		<*0.001*	7.278 [0.521, 14.034]		*0.035*
PKD2	−0.161 [−0.232, −0.090]		<*0.001*	18.476 [11.469, 25.482]		<*0.001*
Constant	2.378 [2.297, 2.458]		<*0.001*	148.405 [139.565, 157.246]		<*0.001*
**B.**	***b***	***t***	***P value***	***b***	***t***	***P value***
PKD1 PT vs. PKD1 ID	−0.02	−0.103	*0.918*	−0.114	−0.671	*0.502*
PKD1 PT vs. PKD1 NT	−0.479	−3.189	*0.002*	0.393	2.891	*0.004*
PKD1 PT vs. PKD2	−0.505	−4.036	<*0.001*	0.428	3.659	<*0.001*
PKD1 PT vs. NM	−0.719	−4.785	<*0.001*	0.622	4.507	<*0.001*
PKD1 ID vs. PKD1 NT	−0.453	−1.032	*0.304*	0.667	1.68	*0.095*
PKD1 ID vs. PKD2	0.035	0.086	*0.931*	0.545	1.524	*0.13*
PKD1 ID vs. NM	−0.713	−1.47	*0.144*	0.718	2.351	*0.02*
PKD1 NT vs. PKD2	0.601	2.476	*0.014*	−0.26	−1.156	*0.249*
PKD1 NT vs. NM	−0.244	−0.936	*0.35*	0.242	1.006	*0.316*
PKD2 vs. NM	−0.874	−3.614	<*0.001*	0.544	2.448	*0.015*

*Adjusted for age and gender.

P value of Log(htTKV) for familial influence = 0.001.

P value of eGFR for familial influence = 0.029.

**Dependent variable = Log(htTKV), eGFR.

By linear regression for verifying moderating effect adjusting for age and gender.

b is the linearity between the two genotypes, and when |b|>1, it is significant; and t is the significance of b coefficient, and when |t| is greater, it is more significant.

Multilevel multivariate linear regression analysis demonstrated that the *PKD1*-PT genotype had a larger htTKV than the *PKD1*-NT or *PKD2* genotypes after adjusting for age, gender, and the familial clustering effect (Table 3). The family-level clustering effect was statistically significant (P = 0.001). The regression model of eGFR showed that the *PKD1*-PT genotype was associated with a lower eGFR than *PKD1*-NT or *PKD2* genotypes (Table 3). The family-level clustering effect was also significant (P = 0.029).

### Incidence of ESRD and death according to PKD genotypes

**Figure 3:**
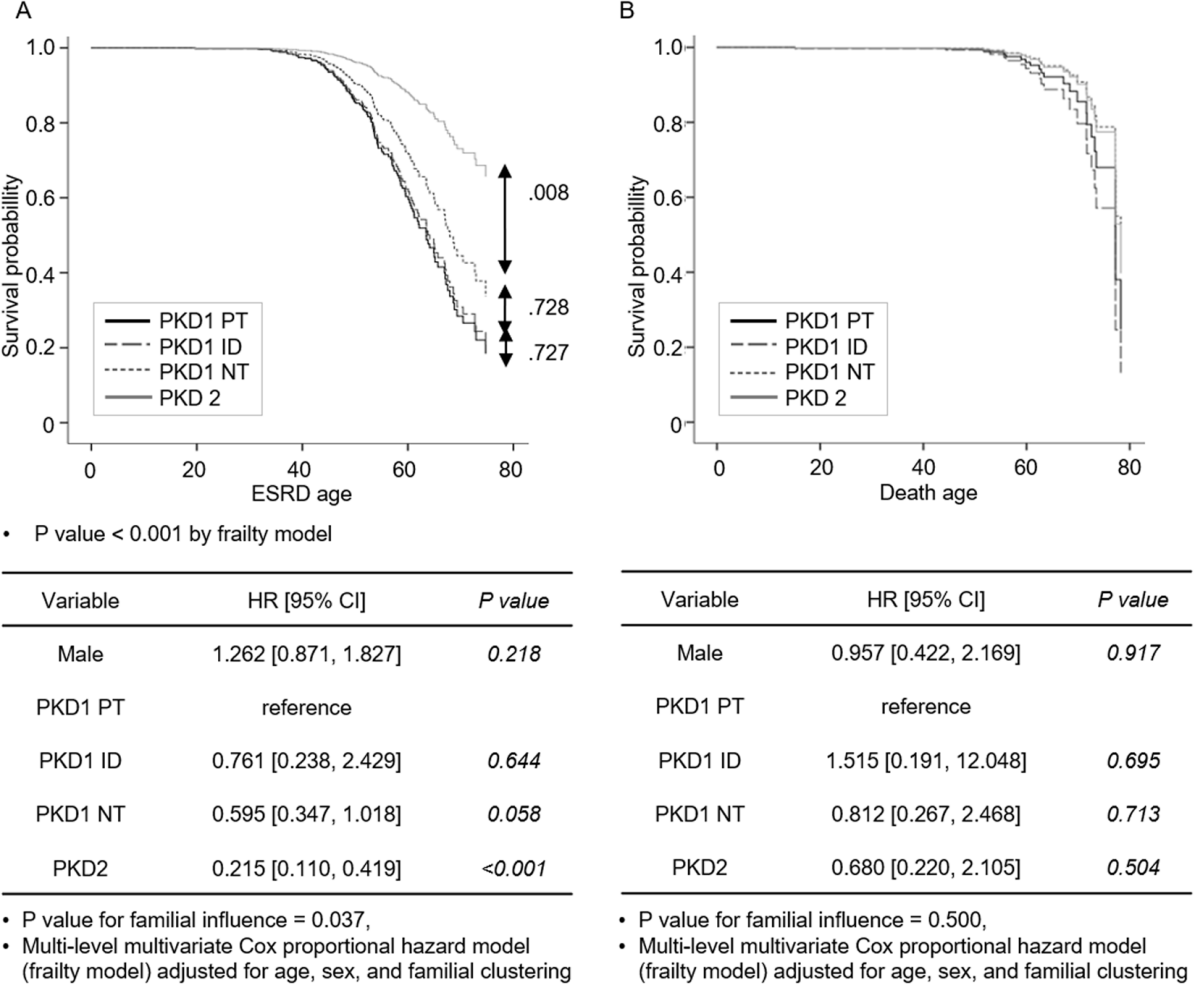
Renal outcomes and death according to type of *PKD1/2* mutation. (**A**) Survival analysis of ESRD progression. (**B**) Survival analysis of all-cause mortality. The median age of ESRD was 63.5 for PKD1-PT genotype, followed by 68.8 for PKD1-NT and 72.9 for PKD2. When *PKD1*-PT genotype was used as a reference, the *PKD2* genotype showed a 0.22 times lower HR of ESRD. The familial clustering effect was significant (P = 0.037). The incidence of death did not have difference according to genotypes. Abbreviations. ESRD, end-stage renal disease; *PKD1*-PT, *PKD1* protein-truncating; *PKD1*-NT, *PKD1* non-truncating; *PKD1*-ID, *PKD1* small in-frameshift indels; HR, hazard ratio; CI, confidence interval.

The *PKD1* genotype showed an earlier onset of ESRD than *PKD2* genotype (64.9 vs. 72.9 years old, P < 0.001) (see Supplementary Fig. S6). Among the 630 patients, with the exclusion of the NM subjects (n = 119) from the total of 749 subjects, 122 ESRD events were observed during follow-up (Table 2). The ESRD incidence was 21.5% (110/511) in *PKD1* and 10.1% (12/119) in *PKD2* (P = 0.003). The incidence of ESRD was lower in the *PKD2* genotype than for other genotypes (*PKD2*, 12 (10.1%) vs. *PKD1*-PT, 84 (22.6%); *PKD1*-ID, 4 (19.0%); *PKD1*-NT, 22 (18.5%), P for trend = 0.004). The median age of ESRD was 63.5 for PKD1-PT genotype, followed by 68.8 for PKD1-NT and 72.9 for PKD2. A multi-level multivariate Cox proportional hazard (frailty) model was used to compare the effect of genotype on ESRD. When *PKD1*-PT genotype was used as a reference, *PKD2* genotype showed a 0.22 times lower hazard ratio than *PKD1*-PT genotype. The familial clustering effect was significant (P = 0.037) (Fig. 3).

There were 23 death events during follow up. Either incidence of death or age of death did not show a significant difference among genotypes (Table 2). In the frailty model, the incidence was too small to find any significant difference according to genotypes (Fig. 3).

## Discussion

As new therapeutic agents became available as disease-course modifying interventions, understanding the natural course of ADPKD and identifying high-risk candidates for new clinical interventions have become important. Although defining genetic variations and their associations with phenotypes is the first step to understand the natural course of genetic diseases, there have been only a few reports on genetic profiles of Asian ADPKD populations^4,10,11,15^.

In this study, we investigated the genetic characteristics of *PKD1/2* mutations using a large prospective Korean ADPKD cohort. In brief, 331 mutations were detected by TES using NGS combined with *PKD1* exon 1 Sanger sequencing and MLPA. The detection rate of mutations was 80.7% (423/524 families). Among them, 70.7% of mutations were novel. The prevalence of *PKD1* and *PKD2* among 524 families was 84% and 16%, respectively. Mutations were more frequently found in the C-type of lectin and GPS domains of polycystin-1 and the linker domain of polycystin-2 among the Korean ADPKD population than among Western populations.

TES using NGS was applied in this study. There was a concern about the decreased efficacy of TES in mutation detection because of large amount of pseudogenic lesion in PKD1. However, demonstrated from the milestone study by Trujillano and colleagues^16^, NGS was not inferior in mutation detection and could be a possible substitute for Sanger sequencing because Sanger sequencing itself is expensive and time consuming. NGS is economically feasible, convenient, and has the possibility of automation. However, low or high GC content can reduce the mutation detection rate by NGS by affecting the probe hybridization and PCR amplification steps. The false negative rate is expected to be elevated for *PKD1* exon 1, which has many GC-rich regions. Moreover, large deletions can easily be missed by NGS. In this study, we tried to compensate for these limitations by exon-specific Sanger sequencing of *PKD1* exon 1 and MLPA. However, the detection rate of in our study was lower than that with Sanger sequencing with long-range PCR^11^. One possible reason for the low detection rate is that we performed target enrichment with whole genomic DNA instead of pure *PKD1* and *PKD2* genomic regions. A previous study suggested using PCR to generate locus-specific amplicons of *PKD1* before sequencing. Therefore, refining the NGS pipeline to increase sequencing depth through locus-specific PCR amplicon enrichment should be considered to improve the detection rate. Another possible explanation is that our population may be enriched with novel Asian-specific missense variants with unknown significance. Therefore, further functional analysis and Sanger sequencing of *PKD1/2* should be considered in the NM cases to further improve the detection rate.

The proportions of *PKD1* and *PKD2* were similar to those from previous data^7^. However, renal survival in *PKD1* carriers was 64.9 years old in Korean ADPKD patients which was approximately 7 years longer than that in Genkyst cohort (58 years old)^8^. More specifically, Korean patients with PKD1-PT genotype showed much better renal survival compared to Genkyst cohort (median age of ESRD 63.5 vs. 55 years old). On the other hands, the median age of ESRD in *PKD2* carriers was 72.9 years old in Korean patients which was approximately 6 years younger than that in Genkyst cohort (79 years old)^8^. This discrepancy may be related to selection bias because our study only included ADPKD patients from tertiary hospitals in Korea. Nonetheless, further study is needed to evaluate genetic and modifiable factors that may influence different renal survival among various ethnicities.

Excluding NM, 331 mutations were ultimately detected in the current study. When compared with those reported in other large cohort studies, the distribution of the mutation classes was similar to those presented in previous studies. Interestingly, our study revealed a high proportion of novel variants. This can be explained by the founder effect or repetitive mutations, as a recent Chinese study also showed similar findings (personal communication).

As part of that effort, we compared the frequency of mutations in certain domains of polycystin-1 or polycystin-2 with the results from the PKDB for the C-type lectin and GPS domains of polycystin-1 and the linker domain of polycystin-2. The C-type lectin domain of polycystin-1 (protein product of *PKD1*) binds carbohydrate matrices *in vitro* and may be involved in protein carbohydrate interactions *in vivo*. Polycystin-1 undergoes cleavage at the juxtamembrane GPS, a process likely to be essential for its biological activity. This GPS is essential for the intracellular transport. Finally, the interaction of polycystin-2 with polycystin-1 is mediated by the C-terminal coiled-coil domain of polycystin-2, and the linker region between the EF-hand and coiled-coil domains is not sufficient to mediate the association by itself. The presence of the N-terminal linker to the polycystin-2 coiled-coil domain allows a tighter association. The implications of these findings for the prognosis for Asian patients with ADPKD should be clarified in the future.

This study enrolled patients with typical ADPKD imaging findings if the subjects had no familial history. Therefore, the NM group seems to be an entity that shares other clinico-genetic characteristics. Cases of NM were not directly included in the analysis, but most of the patients with NM showed similar or milder clinical features compared to *PKD2* genotype, suggesting that they are groups with mutations in regions such as introns or mosaicism, which are difficult to detect using TES. Additionally, Other genes (i.e. GANAB, DNAJB11) involved in atypical presentations of ADPKD could have been part of the targeted NGS approach. Further studies to clarify the meaning of NM are warranted.

We found families that had two or more novel missense variants. In the absence of a functional assay, assessing the putative pathogenicity of missense variants is not reliable. To validate the clinical pathogenicity of missense variants, we analyzed family-based segregation studies including at least one affected and/or one unaffected member in each family. In most of the cases, family-based segregation analysis was very helpful to discriminate the pathogenicity of missense variants. However, for a complete segregation study, it is necessary to investigate all household members accessible through the family tree. Complete segregation can be determined especially clearly when the affected subject is examined together with both parents, which is not always possible. When the affected subject is examined with only his/her siblings or children, the power to completely discriminate the pathogenicity of the variant may be lowered. However, we made every effort to overcome these limitations by enrolling additional families until the pathogenicity of the variants was clearly determined.

Our study showed that *PKD1*-PT genotype was the most common genotype (59%). The distribution of each genotype was similar to what has been reported in previous studies^7^. Subjects with the *PKD1*-PT genotype had an early onset of hypertension, larger htTKV, lower eGFR, and more frequent ESRD incidence than other genotypes. The family-level clustering effect for log(htTKV) and ESRD onset was significant. However, it was also noteworthy that there existed discrepancies between genotypes and phenotypes. For example, the proportions of mild phenotypes of Mayo class 1A and 1B were 7% and 25% in individuals with the *PKD1*-PT genotype, which suggests that other modifying factors may exist.

A strength of the current study is that it is unique as it represents the first large cohort analysis of an Asian population. This study reveals many novel mutations to the *PKD1* and *PKD2* genes as well as novel mutation enrichment sites within the ADPKD proteins. Our study made efforts to overcome the shortcomings of NGS by performing *PKD1* exon-specific Sanger sequencing, MLPA, and a familial segregation study. In addition, our HOPE-PKD cohort is fairly well designed and has a substantial number of subjects. Nonetheless, there are some limitations. Since this study was conducted on patients in tertiary hospitals in South Korea, there is a possibility of selection bias that only severe patients come to the hospital. Thorough evaluation and collection of family history was difficult since not all the members were registered at our hospital. Therefore, it was difficult to investigate the exact family-level clustering effect. Moreover, we could not find familial clustering effects for ESRD or mortality, because of the small number of events. Due to the lost to follow-up of the patient and the absence of DNA samples, we were unable to perform PKD1/PKD2 LR-PCR/Sanger sequencing for the subjects with NM. Therefore, we could not present the exact specificity parameter of NGS. In addition, we used the ellipsoid equation to measure TKV instead of stereology, which is less accurate.

In conclusion, we performed a comprehensive genotype study using one of the largest Asian ADPKD cohorts, HOPE-PKD, and demonstrated the characteristics of variants and mutations in Korean patients. Our results also strongly suggest that an analysis of the ADPKD genotype can contribute to the selection of rapidly progressing patients for new emerging therapeutic interventions.

## Methods

### Subjects

A total of 866 subjects from 641 unrelated families registered in the HOPE-PKD cohort between 2009 and 2016 were screened. All methods were carried out in accordance with relevant guidelines and regulations. The study protocol was approved by the Institutional Review Board of Seoul National University Hospital (IRB No; 1205-112-411). The informed consent was obtained from all subjects before performing study.

Subjects aged over 18 who had compatible imaging findings using the Unified criteria^17^ with or without family history were enrolled in HOPE-PKD cohort. Among the screened patients, 117 patients were excluded from the analysis including those who had active cancer (n = 4) or liver cirrhosis (n = 4) and chronic HBV or HCV hepatitis (n = 18) that may change liver volume or affect renal function and those who had no kidney or liver volume data available (n = 91, see Supplementary Fig. S7). A total of 749 subjects from 524 unrelated families were included in the analysis.

### Data collection

Baseline demographic profiles were collected including age, gender, height, comorbidities (hypertension, diabetes, chronic liver disease, and cancer), familial history of ESRD and their age at the diagnosis of ESRD. Hypertension was considered present when the subject had either systolic blood pressure >140 mmHg or diastolic blood pressure >90 mmHg or was receiving treatment with antihypertensive medications.

Serum creatinine was measured using the Jaffe method and traced using isotope dilution mass spectrometry. The Chronic Kidney Disease Epidemiology Collaboration formula was used to calculate eGFR^18^. ESRD was defined as either an eGFR <15 mL/min/1.73 m^2^ or initiation of renal replacement therapy. Participants underwent non-contrast computed tomography (CT) exams every 2 to 3 years using multi-detector CT scanners. TKV was measured by the ellipsoid method and adjusted to height^19^ by a single skilled technician.

### Variant screening of *PKD1* and *PKD2* by TES

Genetic analysis of the *PKD1* and *PKD2* genes was performed using TES. For cases with inadequate results, additional Sanger sequencing for exon 1 of *PKD1* gene and MLPA were done. For determining pathogenicity, familial segregation analysis was performed.

For TES, a customized 120-mer RNA bait was designed to capture all exons of the *PKD1* and *PKD2* genes and their flanking intronic sequences^16^ (see Supplementary Tables S3 and S4). The bait was tiled 3 times to increase the coverage of poorly covered regions. For the generation of standard target-all exon capture libraries, the Agilent SureSelect™ target enrichment protocol for the Illumina paired-end sequencing library (version B.3, June 2015) was used together with an input of 500 ng of genomic DNA and sequenced using the HiSeq™ 2500 platform (Illumina, San Diego, CA, USA). In all cases, the SureSelectXT Custom probe set was used.

Sequencing reads were first aligned with the human genome reference sequence (hg19) using BWA version 0.7.5a with the MEM algorithm (default options). To minimize false-positive and false-negative calls, we only used uniquely mapped reads that were properly paired to avoid mapping reads that aligned to both target regions and pseudogenes. In addition, only selected reads with a high mapping quality (>30) were used. SAMTOOLS version 1.2 and Picard version 1.127 (http://picard.sourceforge.net) were used to process SAM/BAM files to duplicate the marking. Specifically, local realignment to reduce any misalignment in the duplicated regions was performed.

We used RealignerTargetCreator and IndelRealigner from GATK version 3.3-1 with known single-nucleotide polymorphisms (SNPs) and indels from dbSNP142, Mills and 1000 G gold-standard indels at hg19 sites, and 1000 G phase 1 indels at hg19 sites. Known SNPs and indels were also used to perform a base calibration. For calling variants, Unified Genotyper was used, and the called variants were recalibrated by GATK based on dbSNP142, Mills indels, HapMap, and Omni. ANNOVAR was used to annotate the variants. The depth of coverage approach was applied to detect any large rearrangement or copy number variations. The CalculateHsMetrics module in PICARD was used to calculate the statistics of each parameter of the samples and depth of each exon, and the copy number ratio was characterized by the normalized depth of the exons from all the patients (see Supplementary Fig. S1).

### Prioritization of detected variants

To identify causal variants, we first selected exonic and splicing variants including nonsynonymous variants and small indels. Variants with an allele frequency over 1% were discarded based on NHLBI-ESP 6500, the 1000 Genome Project, and an in-house database consisting of exomes of 192 (Samsung Medical Center, Korea) and 397 (Korean Bioinformation Center, Korea) normal Korean individuals. Variants that were not reported in dbSNP142 were included, and those with low-quality reads (<20) and low genotype quality (<30) were excluded (see Supplementary Fig. S1).

The detected variants were classified into seven categories of nonsense, frameshift, typical splicing, large deletion, missense, small in-frame indel and NV. Seven classes of variants were further divided into five grades according to their pathogenicity: DP, HLP, LP, LN, and I (see Supplementary Fig. S8). The DP variants were defined as all the variants that result in protein truncation, including nonsense, frameshift, typical splicing, and large deletion variants or those previously established as DP in PKDB or previous articles. The HLP variants were defined as missense variants or small in-frame indels that were previously established as HLP in PKDB or previous articles. The LP variants were defined as missense variants or small in-frame indels that were previously established as LP in PKDB or previous articles or variants that were predicted to be damaging using in-silico analysis and segregated within a family. Variants were thought to be damaging if the results of in-silico analysis satisfied two of the following conditions: 1) damaging as predicted by a SIFT score ≤ 0.05, 2) damaging as predicted by Polyphen-2 (HumDiv), and 3) GERP++ score ≥ 4. The definitions of grades are described in the Supplementary Table S7.

For genotype-phenotype analysis, *PKD1* or *PKD2* mutations were further classified into four PKD genotypes according to the previously described manner^14^; *PKD1*-PT, *PKD1*-ID, *PKD1*-NT, and *PKD2* genotypes.

### Validation of variants detected by TES using LR-PCR/Sanger sequencing

The validation set (n = 80) was developed to confirm the variants that was discovered by NGS approach by long-range PCR/Sanger sequencing. The pathogenic variants detected by TES were confirmed by direct sequencing of the corresponding gene regions using the ABI3730xl Genetic Analyzer (Applied Biosystems, Foster City, CA). For the duplicated part of *PKD1*, long-range PCR followed by nested-PCR was performed using *PKD1*-specific primers.

### Improving mutation detection rate by *PKD1* exon 1-specific sanger sequencing and MLPA

The subjects with LN or I variants or those in whom NV was detected underwent Sanger sequencing of *PKD1* exon 1 and MLPA. Firstly, Sanger sequencing of *PKD1* exon 1 was performed because variants might not be detected by NGS owing to low coverage of exon 1 (see Supplementary Fig. S9). For the duplicated part of *PKD1*, long-range PCR followed by nested PCR was performed using *PKD1*-specific primers as described before with minor modifications^20,21^. In brief, 300–400 ng of genomic DNA was amplified in a final volume of 50 μL, containing, 0.2 μmol/L of each primer (Cosmogentech, Korea), 0.5 mol/L betaine (Sigma, USA), 5% dimethyl sulfoxide (Sigma, USA) in the AccuPower ProFi Taq PCR Premix (Bioneer, Korea) as a Touchdown protocol. The long-range PCR products were purified with the Qiaquick PCR purification kit and were further amplified using a nested PCR. The long-range PCR for the *PKD1* exon was done using the AccuPower HotStart PCR Premix (Bioneer, Korea). Sanger sequencing was performed using the ABI3730xl Genetic Analyzer (Applied Biosystems, Foster City, CA, USA). In addition, MLPA was performed to detect large deletion cases if both TES and *PKD1* exon 1 sequencing revealed NM. MLPA analysis was performed with a SALSA MLPA KIT P351-B1/P352-B1 *PKD1*-*PKD2* kit (MRC-Holland, Amsterdam, the Netherlands) according to the manufacturer’s instructions^12^ and single probe exon deletions via MLPA was confirmed by direct sequencing that they are not due to SNP under the probe (for the primer, see Supplementary Table S8). Finally, we looked into 7 variants found through MLPA. Our read depth did not show any particular problems for the cases. However, only 3 out of 6 variants were detected. The normalized read depth in *PKD1* and *PKD2* of mutations that detected via MLPA was shown in Supplementary Fig. S10. It was not a matter of read depth but a technical limitation of NGS approach in the case of large deletion.

### Familial segregation analysis

When a novel LP variant appeared after variant calling, familial segregation analysis was performed to confirm pathogenicity of the variant. Family members of the subject with LP variant were additionally enrolled for familial segregation study. The parents of the subjects were recruited first. If the parents were absent, siblings with and without ADPKD were recruited for segregation analysis. The presence or absence of ADPKD was screened with portable sonography (SONON 300 C, Healcerion, Korea), and additional CT scans were performed in non-diagnostic cases. For familial segregation analysis, we utilized both direct Sanger sequencing and TES.

### Statistical analysis

Statistical analyses were performed using SPSS version 23.0 (IBM Corp., Armonk, NY, USA). Analysis of covariance, the Mann-Whitney test, and the chi-square test were performed to compare variables between two groups. *P* values < 0.05 were considered statistically significant. A multilevel linear regression model in Stata 14 (StataCorp, College Station, TX, USA) was performed to identify the correlations between htTKV or eGFR and PKD genotypes. To control the clustering effect, variance estimation was also done by the clustered sandwich estimator. For survival analysis, multivariate analysis using a multilevel Cox proportional hazard model (frailty model) adjusting for age, gender, and the family-level clustering effect was performed to determine risk factors for ESRD or mortality. The linearity assumption of continuous variables was verified, and proportional hazards assumption of categorical variables was verified using a log-minus-log plot.

## Supplementary information

Supplementary information

## Data Availability

All produced data are available as Supplementary Information online.
