# Author Correction: Genetic Characteristics of Korean Patients with Autosomal Dominant Polycystic Kidney Disease by Targeted Exome Sequencing

**DOI:** 10.1038/s41598-020-62489-8

**Published:** 2020-04-02

**Authors:** Hyunsuk Kim, Hayne Cho Park, Hyunjin Ryu, Hyunho Kim, Hyun-Seob Lee, Jongho Heo, Chung Lee, Nayoung K. D. Kim, Woong-Yang Park, Young-Hwan Hwang, Kyu Beck Lee, Kook-Hwan Oh, Yun kyu Oh, Curie Ahn

**Affiliations:** 1 Department of Internal Medicine, Chuncheon Sacred Heart Hospital, Chuncheon, Korea; 2 Department of Internal Medicine, Kangnam Sacred Heart Hospital, Seoul, Korea; 3 Department of Internal Medicine, Seoul National University Hospital, Seoul, Korea; 4 Center for Medical Innovation, Seoul National University Hospital, Seoul, Korea; 5 Genomic Core Facility, Transdisciplinary Research and Collaboration Division, Translational Research Institute, and Biomedical Research Institute, Seoul National University Hospital, Seoul, Korea; 6 National Assembly Futures Institute, Seoul, Korea; 7 Samsung Genome Institute, Samsung Medical Center, Seoul, Korea; 8 Department of Health Science and Technology, Samsung Advanced Institute for Health Sciences and Technology, Sungkyunkwan University, Seoul, Korea; 9 Department of Molecular Cell Biology, Sungkyunkwan University School of Medicine, Seoul, Korea; 10 Truewords Dialysis Clinic, Incheon, Korea; 11 Department of Internal Medicine, Kangbuk Samsung Hospital, Sungkyunkwan University School of Medicine, Seoul, Korea; 12 Department of Internal Medicine, Seoul National University Boramae Medical Center, Seoul, Korea

Correction to: *Scientific Reports* 10.1038/s41598-019-52474-1, published online 18 November 2019

The Supplementary Information file accompanying this Article contains errors.

In Supplementary Table S6A, ‘PKD1 protein-truncating mutations in study families’, the protein change for Family ID ‘5, 117’,

“p.Gly3808X”

should read:

“p.Trp3808X”

In the same table, the cDNA change for Family ID ‘213’,

“c.10429-2_10429-1delCA”

should read:

“c.10220+2_10220+3del”

In addition, in Supplementary Table S6C, ‘PKD1 non-truncating mutations in study families’, the protein change for Family ID ‘304’,

“p.Gly381Arg”

should read:

“p.Gly381Ser”

