# Supplementary material for: Genetic Characteristics of Korean Patients with Autosomal Dominant Polycystic Kidney Disease by Targeted Exome Sequencing
Source: Sci Rep. 2019 Nov 18;9:16952. doi: 10.1038/s41598-019-52474-1 (PMC6861305; doi:10.1038/s41598-019-52474-1)
Supplement: Supplementary file 1 — Supplementary information [file 41598_2019_52474_MOESM1_ESM.pdf]

Genetic Characteristics of Korean Patients with Autosomal Dominant Polycystic Kidney Disease by Targeted Exome Sequencing

Hyunsuk Kim[1], Hayne Cho Park[2], Hyunjin Ryu[3], Hyunho Kim[4], Hyun-Seob Lee[5], Jongho Heo[6], Chung Lee[7], Nayoung K.D. Kim[7], Woong-Yang Park[7,8,9], Young-Hwan Hwang[10], Kyu Beck Lee[11], Kook-Hwan Oh[3], Yun kyu Oh[*12], Curie Ahn[*3]

Supplementary Table S1. Demographic Findings of 749 Patients (524 Families)

Variable	Total	Male	Female	*P* value
N (%)	749	360 (48.1)	389 (51.9)	
Age [mean±SD]	46.4±13.3	44.6±14.4	48.0±12.0	<0.001
Age groups, n(%)				
~30	88 (11.8)	62 (17.2)	26 (6.7)	0.001
30~40	149 (19.9)	69 (19.2)	80 (20.6)	
40~50	210 (28.0)	93 (25.8)	117 (30.1)	
50~60	183 (24.4)	86 (23.9)	97 (24.9)	
60~70	95 (12.7)	41 (11.4)	54 (13.9)	
70~	24 (3.2)	9 (2.5)	15 (3.9)	
eGFR, [mean±SD]	65.8±38.9	65.6±39.4	66.0±38.5	0.896
CKD stages, n(%)				0.945
Stage I	233 (32.6)	107 (31.1)	126 (34.0)	
Stage II	198 (27.7)	96 (26.7)	102 (27.5)	
Stage IIIa	85 (11.9)	42 (11.7)	43 (11.6)	
Stage IIIb	40 (5.6)	21 (5.8)	19 (5.1)	
Stage IV	22 (3.1)	12 (3.3)	10 (2.7)	
Stage V	137 (19.2)	66 (18.3)	71 (19.1)	

eGFR of subjects with RRT or kidney transplantation was regarded as 15 mL/min/1.73 m^2.

Abbreviations. SD, standard deviation; eGFR, estimated glomerular flow rate

Supplementary Table S2. Results from Validation Study (n=80)

Gene	ExonicFunc	Exon	cDNA change	Protein change	DupR	Sanger
PKD1	frameshift deletion	exon15	c.5014_5015del	p.Arg1672GlyfsX98	Y	Confirmed
PKD1	frameshift insertion	exon16	c.6994_7000dup	p.Val2334GlyfsX88	Y	Confirmed
PKD1	stopgain SNV	exon15	c.4447C>T	p.Gln1483X	Y	Confirmed
PKD1	stopgain SNV	exon15	c.6549dup	p.Glu2184X	Y	Confirmed
PKD1	frameshift deletion	exon25	c.9083_9084del	p.Glu3028GlyfsX40	Y	Confirmed
PKD1	stopgain SNV	exon15	c.4447C>T	p.Gln1483X	Y	Confirmed
PKD1	stopgain SNV	exon20	c.7816C>T	p.Gln2606X	Y	Confirmed
PKD1	frameshift deletion	exon15	c.5014_5015del	p.Arg1672GlyfsX98	Y	Confirmed
PKD1	frameshift deletion	exon15	c.4070del	p.Leu1357ArgfsX9	Y	Confirmed
PKD1	frameshift insertion	exon11	c.2494dupC	p.Arg832ProfsX40	Y	Confirmed
PKD1	stopgain SNV	exon15	c.4447C>T	p.Gln1483X	Y	Confirmed
PKD1	stopgain SNV	exon5	c.1198C>T	p.Arg400X	Y	Confirmed
PKD1	frameshift deletion	exon11	c.2618_2621del	p.Val873AlafsX24	Y	Confirmed
PKD1	frameshift deletion	exon15	c.5014_5015del	p.Arg1672GlyfsX98	Y	Confirmed
PKD1	NA	exon23	c.8017-2A>G		Y	Confirmed
PKD1	stopgain SNV	exon15	c.4447C>T	p.Gln1483X	Y	Confirmed
PKD1	stopgain SNV	exon15	c.6549dup	p.Glu2184X	Y	Confirmed
PKD2	stopgain SNV	exon13	c.2407C>T	p.Arg803X	N	Confirmed
PKD1	frameshift deletion	exon15	c.3684del	p.Val1229TrpfsX44	Y	Confirmed
PKD1	stopgain SNV	exon15	c.6549dup	p.Glu2184X	Y	Confirmed
PKD1	nonsynonymous SNV	exon23	c.8311G>A	p.Glu2771Lys	Y	Confirmed
PKD1	nonframeshift deletion	exon24	c.8935_8937del	p.Phe2979del	Y	Confirmed
PKD1	nonsynonymous SNV	exon27	c.9404C>T	p.Thr3135Met	Y	Confirmed
PKD1	nonsynonymous SNV	exon15	c.4051C>T	p.Arg1351Trp	Y	Confirmed
PKD1	stopgain SNV	exon15	c.3334G>T	p.Glu1112X	Y	Confirmed
PKD1	frameshift insertion	exon15	c.3349dup	p.Gln1117ProfsX19	Y	Confirmed
PKD1	stopgain SNV	exon9	c.1789C>T	p.Gln597X	Y	Confirmed

PKD1	frameshift insertion	exon15	c.4315_4324delinsCAACTGTTG	p.Gly1439GlnfsX5	Y	Confirmed
PKD1	frameshift deletion	exon7	c.1495del	p.Glu499SerfsX59	Y	Confirmed
PKD1	frameshift deletion	exon11	c.2659del	p.Trp887GlyfsX11	Y	Confirmed
PKD1	frameshift deletion	exon15	c.4679_4691del	p.Val1560GlyfsX3	Y	Confirmed
PKD1	frameshift deletion	exon46	c.12672_12673del	p.Gln4224HisfsX2	N	Confirmed
PKD1	frameshift deletion	exon15	c.3744_3754del	p.Asp1249AlafsX48	Y	Confirmed
PKD1	frameshift deletion	exon19	c.7579_7580del	p.Val2527LeufsX67	Y	Confirmed
PKD1	frameshift deletion	exon11	c.2659del	p.Trp887GlyfsX11	Y	Confirmed
PKD1	stopgain SNV	exon15	c.5477G>A	p.Trp1826X	Y	Confirmed
PKD1	frameshift deletion	exon8	c.1669_1670del	p.Leu557AspfsX29	Y	Confirmed
PKD1	frameshift deletion	exon11	c.2716del	p.Glu906SerfsX21	Y	Confirmed
PKD1	frameshift deletion	exon23	c.8327_8330del	p.Leu2776ArgfsX98	Y	Confirmed
PKD1	stopgain SNV	exon17	c.7164C>G	p.Tyr2388X	Y	Confirmed
PKD1	stopgain SNV	exon17	c.7164C>G	p.Tyr2388X	Y	Confirmed
PKD1	frameshift deletion	exon15	c.6808_6811del	p.Asp2270HisfsX43	Y	Confirmed
PKD1	frameshift deletion	exon21	c.7973_7974del	p.Val2658GlyfsX2	Y	Confirmed
PKD1	nonframeshift deletion	exon20	c.7837_7839del	p.Leu2613del	Y	Confirmed
PKD1	stopgain SNV	exon5	c.696T>A	p.Cys232X	Y	Confirmed
PKD1	stopgain SNV	exon3	c.350T>G	p.Leu117X	Y	Confirmed
PKD1	stopgain SNV	exon5	c.706C>T	p.Gln236X	Y	Confirmed
PKD1	frameshift deletion	exon8	c.1669_1670del	p.Leu557AspfsX29	Y	Confirmed
PKD1	stopgain SNV	exon15	c.4797C>A	p.Tyr1599X	Y	Confirmed
PKD1	stopgain SNV	exon15	c.5477G>A	p.Trp1826X	Y	Confirmed
PKD1	frameshift deletion	exon23	c.8327_8330del	p.Leu2776ArgfsX98	Y	Confirmed
PKD1	nonframeshift deletion	exon23	c.8308_8310del	p.Asn2770del	Y	Confirmed
PKD1	stopgain SNV	exon6	c.1316G>A	p.Trp439X	Y	Confirmed
PKD1	frameshift insertion	exon16	c.7003dupG	p.Glu2335GlyfsX85	Y	Confirmed
PKD1	frameshift deletion	exon14	c.3223_3226del	p.Phe1075ArgfsX28	Y	Confirmed

PKD1	frameshift insertion	exon15	c.5517_5521dup	p.Val1841GlyfsX110	Y	Confirmed
PKD1	stopgain SNV	exon36	c.10809G>A	p.Trp3603X	N	Confirmed
PKD1	stopgain SNV	exon15	c.4861C>T	p.Gln1621X	Y	Confirmed
PKD2	stopgain SNV	exon1	c.547C>T	p.Gln183X	N	Confirmed
PKD2	frameshift deletion	exon11	c.2140delA	p.Lys714AsnfsX2	N	Confirmed
PKD1	stopgain SNV	exon15	c.3607C>T	p.Gln1203X	Y	Confirmed
PKD2	stopgain SNV	exon1	c.547C>T	p.Gln183X	N	Confirmed
PKD1	stopgain SNV	exon15	c.6561G>A	p.Trp2187X	Y	Confirmed
PKD1	frameshift deletion	exon20	c.7816del	p.Gln2606SerfsX14	Y	Confirmed
PKD1	frameshift deletion	exon44	c.12100del	p.Val4034CysfsX4	N	Confirmed
PKD1	frameshift deletion	exon37	c.10839_10840del	p.Phe3614LeufsX11	N	Confirmed
PKD1	nonsynonymous SNV	exon36	c.10772C>T	p.Ser3591Phe	N	Confirmed
PKD1	nonframeshift deletion	exon10	c.2065_2067del	p.Ser689del	Y	Confirmed
PKD1	nonframeshift deletion	exon10	c.2065_2067del	p.Ser689del	Y	Confirmed
PKD1	nonsynonymous SNV	exon2	c.242C>T	p.Ala81Val	Y	Confirmed
PKD1	nonsynonymous SNV	exon11	c.2722G>A	p.Val908Met	Y	Confirmed
PKD1	nonsynonymous SNV	exon10	c.2081C>T	p.Pro694Leu	Y	Not detected
PKD1	nonframeshift deletion	exon10	c.2065_2067del	p.Ser689del	Y	Confirmed
PKD1	nonsynonymous SNV	exon15	c.4955T>C	p.Leu1652Pro	Y	Confirmed
PKD1	nonframeshift deletion	exon25	c.9092_9094del	p.Leu3031del	Y	Confirmed
PKD1	nonsynonymous SNV	exon2	c.242C>T	p.Ala81Val	Y	Confirmed
PKD1	nonframeshift insertion	exon15	c.6258_6263dup	p.Pro2087_Arg2088dup	Y	Confirmed
PKD1	nonframeshift deletion	exon25	c.9092_9094del	p.Leu3031del	Y	Confirmed
PKD1	nonsynonymous SNV	exon25	c.9128G>A	p.Cys3043Tyr	Y	Confirmed
PKD1	nonsynonymous SNV	exon45	c.12313A>C	p.Ile4105Leu	N	Confirmed

Supplementary Table S3. Design of Custom Bait

Category	Description
Sequencing technology	Illumina
Sequencing protocol	Paired-End Short Read (100bp)
Tiling frequency	3x
Bait length	120
Avoid standard repeat masked regions	Yes
Avoid overlap	20
Layout strategy	Centered
Strand	Sense
Total input targets	1018
Total valid targets	256
Average number of baits per target	5.88
Total targets with bait coverage	2038
Total number of baits	1498
Baits removed due to avoid overlap	46
Total baits covered by baits	59913

Abbreviations. bp, basepair

Supplementary Table S4. Quality of Targeted Exome Sequencing

Cohort (N=749)	Average	S.D.
QC passed reads	9141842.96	6008387.66
Unique reads	7826567.35	5238200.17
Aligned unique reads	7815828.12	5227207.04
PKD1 target mean coverage (X)	1523.12	973.96
% of PKD1 target bases ≥1X	0.9931	0.0076
% of PKD1 target bases ≥10X	0.9700	0.0073
% of PKD1 target bases ≥20X	0.9650	0.0061
% of PKD1 target bases ≥50X	0.9587	0.0084
% of PKD1 target bases ≥100X	0.9478	0.0207
PKD2 target mean coverage (X)	1422.98	903.62
% of PKD2 target bases ≥1X	0.9999	0.0009
% of PKD2 target bases ≥10X	0.9960	0.0074
% of PKD2 target bases ≥20X	0.9879	0.0158
% of PKD2 target bases ≥50X	0.9594	0.0297
% of PKD2 target bases ≥100X	0.9226	0.0447

Abbreviations. QC, quality control; S.D, standard deviation

Supplementary Table S5. Mutation frequency between different ADPKD cohort

Gene/Mutation Type	HALT (n, pedigree)	%	GENKYST (n, pedigree)	%	HOPE-PKD (n, pedigree)	%
**PKD1**	731	84.4	392	80.5	348	82.3
**PKD1-PT**	478	55.2	255	52.4	246	58.2
**PKD1-NT**	253	29.2	137	28.1	102	24.1
**PKD2**	135	15.6	95	19.5	75	17.7
**Total**	866	100	487	100	423	100

Supplementary Table S6. Mutations found in this study

Supplemental Table S6A. PKD1 protein-truncating mutations in study families

Family ID	Exon	Codon	cDNA change	Protein change	Predicted effect	Domain	PKDB	Canada DB	Korean individuals	Korean families
716	1	5	c.13_25del	p.Ala5TrpfsX53	Frameshift	LRR	1	0	1	1
61	1	9	c.25_26dup	p.Ala9TrpfsX64	Frameshift	Extracellular sequence	0	0	1	1
126	1	15	c.43_62del	p.Leu15AlafsX92	Frameshift	Extracellular sequence	0	0	3	1
57	1	33	c.78_96dup	p.Cys33ArgfsX87	Frameshift	LRR	0	0	1	1
473, 886	1	44	c.129_135delCGGCGCC	p.Gly44ProfsX27	Frameshift	LRR	0	0	2	2
904	1	48	c.142del	p.Arg48AlafsX25	Frameshift	LRRCT	0	0	1	1
260	1	56	c.165_171delGCTGCGG	p.Leu56ArgfsX15	Frameshift	LRR	1	0	1	1
185	4	123	c.369del	p.Ser123ArgfsX167	Frameshift	Extracellular sequence	0	0	1	1
224	4	142	c.423_424del	p.Glu142AlafsX36	Frameshift	LRRCT	0	0	2	1
415	5	275	c.822_832del	p.Ala275GlyfsX92	Frameshift	PKD 1	0	0	1	1
181, 478, 657	5	286	c.856_862del	p.Gly286X	Frameshift	PKD 1	1	3x	3	3
570	6	442	c.1324del	p.Ala442ProfsX23	Frameshift	C-type lectin	0	0	1	1
16	7	499	c.1495del	p.Glu499SerfsX59	Frameshift	C-type lectin	0	0	3	1
67	8	557	c.1669_1670del	p.Leu557AspfsX29	Frameshift	Extracellular sequence	0	0	1	1
649	9	592	c.1776del	p.Glu592AsnfsX192	Frameshift	Extracellular sequence	0	0	1	1
599	10	630	c.1889dup	p.Asp630GlyfsX83	Frameshift	Extracellular sequence	0	0	1	1

229	10	668	c.2001dup	p.Gly668TrpfsX46	Frameshift	LDL-receptor class A; aTypical	0	0	2	1
363	10	681	c.2040dup	p.Ala681CysfsX33	Frameshift	Extracellular sequence	0	0	1	1
302, 414	10	696	c.2085dup	p.Ala696ArgfsX18	Frameshift	Extracellular sequence	0	0	2	2
793	11	797	c.2390dup	p.Val797GlyfsX19	Frameshift	PKD 2	0	0	1	1
611	11	824	c.2470del	p.Leu824CysfsX74	Frameshift	Extracellular sequence	0	0	2	1
79, 204	11	832	c.2494dup	p.Arg832ProfsX40	Frameshift	Extracellular sequence	0	0	2	2
252	11	873	c.2618_2621del	p.Val873AlafsX24	Frameshift	PKD 3	1	1x	5	1
20	11	887	c.2659del	p.Trp887GlyfsX11	Frameshift	PKD 3	0	0	12	1
73	11	906	c.2716del	p.Glu906SerfsX21	Frameshift	PKD 3	0	0	2	1
110	11	928	c.2784_2796del	p.Glu928ValfsX18	Frameshift	PKD 3	0	0	1	1
541	12	955	c.2865del	p.Val955TrpfsX20	Frameshift	PKD 4	1	0	1	1
318	12	966	c.2896del	p.Arg966GlyfsX10	Frameshift	PKD 4	0	0	1	1
197	13	1029	c.3085_3088del	p.Ala1029CysfsX8	Frameshift	PKD 5	0	0	1	1
146	14	1075	c.3223_3226del	p.Phe1075ArgfsX28	Frameshift	PKD 5	0	0	1	1
54	15	1108	c.3323del	p.Ser1108LeufsX7	Frameshift	PKD 5	0	0	5	1
4	15	1117	c.3349dup	p.Gln1117ProfsX19	Frameshift	PKD 5	0	0	3	1
748	15	1168	c.3503_3504del	p.Pro1168ArgfsX42	Frameshift	PKD 6	1	0	1	1
900	15	1174	c.3520_3521del	p.Gln1174AlafsX36	Frameshift	PKD 6	0	0	1	1
245	15	1205	c.3613_3622del	p.Asp1205SerfsX12	Frameshift	PKD 6	0	0	3	1

160	15	1228	c.3684del	p.Val1228TrpfsX44	Frameshift	PKD 7	1	0	2	1
47	15	1248	c.3744_3754del	p.Asp1248AlafsX48	Frameshift	PKD 7	0	0	2	1
265	15	1303	c.3906dup	p.Ala1303ArgfsX8	Frameshift	PKD 8	0	0	1	1
209	15	1347	c.4041_4042del	p.His1347GlnfsX83	Frameshift	PKD 8	1	X1	1	1
365, 887	15	1357	c.4069del	p.Leu1357TrpfsX9	Frameshift	PKD 8	1	0	2	2
76	15	1357	c.4070del	p.Leu1357ArgfsX9	Frameshift	PKD 8	1	0	5	1
15	15	1439	c.4315_4324delinsCAA CTGTTG	p.Gly1439GlnfsX5	Frameshift	PKD 9	0	0	3	1
279	15	1460	c.4379_4380del	p.Val1460GlyfsX62	Frameshift	PKD 9	0	0	1	1
368	15	1467	c.4401_4407del	p.Val1467AlafsX64	Frameshift	PKD 9	0	0	1	1
369	15	1478	c.4434del	p.Leu1478TrpfsX55	Frameshift	PKD 8	0	0	1	1
888	15	1529	c.4586del	p.Gly1529AlafsX5	Frameshift	PKD 10	0	0	1	1
732	15	1551	c.4650dup	p.Leu1551AlafsX27	Frameshift	PKD 11	0	0	1	1
21	15	1560	c.4679_4691del	p.Val1560GlyfsX3	Frameshift	PKD 11	0	0	4	1
199	15	1599	c.4797del	p.Tyr1599X	Frameshift	PKD 11	0	novel	1	1
500	15	1642	c.4924del	p.Arg1642AlafsX80	Frameshift	REJ	0	0	1	1
1, 33, 336, 457, 542, 562, 575, 635, 666	15	1672	c.5014_5015del	p.Arg1672GlyfsX98	Frameshift	PKD 12	1	29x	17	9
147, 553	15	1841	c.5517_5521dup	p.Val1841GlyfsX110	Frameshift	PKD 14	0	0	2	2
901	15	1877	c.5629del	p.Ala1877ProfsX72	Frameshift	PKD14	0	0	1	1
582	15	2075	c.6224_6225insGTTG	p.Ser2075LeufsX6	Frameshift	PKD 11	0	0	1	1

364	15	2114	c.6341_6344del	p.Tyr2114X	Frameshift	PKD 17	0	0	1	1
238	15	2183	c.6549_6550del	p.Glu2183ValfsX77	Frameshift	REJ	0	0	3	1
215	15	2257	c.6770dup	p.Pro2257AlafsX4	Frameshift	REJ	0	0	1	1
84	15	2270	c.6808_6811del	p.Asp2270HisfsX43	Frameshift	REJ	1	0	1	1
376	16	2332	c.6994_7000del	p.Ala2332TrpfsX7	Frameshift	REJ	1	0	1	1
17	16	2334	c.6994_7000dup	p.Val2334GlyfsX88	Frameshift	REJ	1	0	6	1
136	16	2335	c.7003dup	p.Glu2335GlyfsX85	Frameshift	REJ	0	0	2	1
232	18	2449	c.7345dup	p.Thr2449AsnfsX52	Frameshift	REJ	0	0	1	1
288	18	2470	c.7408_7414del	p.Pro2470TrpfsX148	Frameshift	REJ	0	0	1	1
60	19	2527	c.7579_7580del	p.Val2527LeufsX67	Frameshift	REJ	0	0	1	1
183	19	2542	c.7625del	p.Gly2542ValfsX78	Frameshift	REJ	0	0	1	1
285	20	2581	c.7741_7742dup	p.Thr2581GlnfsX39	Frameshift	REJ	0	0	1	1
157, 655	20	2606	c.7816del	p.Gln2606SerfsX14	Frameshift	REJ	0	0	4	2
559	21	2648	c.7942del	p.Glu2648ArgfsX6	Frameshift	REJ	0	0	1	1
85	21	2658	c.7973_7974del	p.Val2658GlyfsX2	Frameshift	REJ	0	0	2	1
277, 539	22	2674	c.8019dup	p.Pro2674AlafsX148	Frameshift	REJ	0	0	2	2
78, 128	23	2776	c.8327_8330del	p.Leu2776ArgfsX98	Frameshift	REJ	0	0	4	2
551	23	2800	c.8399del	p.Pro2800GlnfsX75	Frameshift	REJ	0	0	1	1
315	23	2859	c.8570_8574dup	p.Ala2859ProfsX18	Frameshift	Extracellular sequence	0	0	1	1

592	23	2861	c.8581dup	p.Ile2861AsnfsX76	Frameshift	Extracellular sequence	0	0	1	1
586	23	2881	c.8642_8655del	p.Asp2881GlyfsX51	Frameshift	Extracellular sequence	0	0	1	1
556, 589, 746	24	2951	c.8851dup	p.Arg2951ProfsX4	Frameshift	Extracellular sequence	0	0	3	3
49, 789	25	3028	c.9083_9084del	p.Glu3028GlyfsX40	Frameshift	GPS	1	0	2	2
754	26	3080	c.9240_9241del	p.Ala3080CysfsX96	Frameshift	Transmembrane	0	0	1	1
397	28	3228	c.9683dup	p.Leu3228ProfsX24	Frameshift	PLAT	0	0	1	1
425	29	3262	c.9780_9784dup	p.Ile3262SerfsX56	Frameshift	Cytoplasmic sequence	0	0	1	1
881	29	3281	c.9841del	p.Ala3281ProfsX35	Frameshift	Cytoplasmic sequence	0	0	1	1
627	29	3281	c.9843del	p.Thr3281ProfsX34	Frameshift	Cytoplasmic sequence	0	0	1	1
407	31	3382	c.10144dup	p.Thr3382AsnfsX8	Frameshift	Cytoplasmic sequence	0	0	1	1
766	31	3388	c.10162_10163dup	p.Glu3388LeufsX8	Frameshift	Cytoplasmic sequence	0	0	1	1
118	33	3419	c.10255dup	p.Trp3419LeufsX7	Frameshift	Cytoplasmic sequence	0	0	2	1
107	35	3529	c.10585_10586dup	p.Gln3529HisfsX56	Frameshift	Cytoplasmic sequence	0	0	1	1
39	36	3569	c.10706_10707del	p.Val3569GlyfsX56	Frameshift	Transmembrane	0	0	2	1
119	36	3581	c.10742del	p.Pro3581ArgfsX3	Frameshift	Extracellular sequence	1	0	2	1
161	37	3613	c.10836_10837del	p.Tyr3613LeufsX12	Frameshift	Cytoplasmic sequence	0	0	1	1
96	37	3655	c.10963del	p.Leu3655TrpfsX28	Frameshift	Cytoplasmic sequence	1	0	1	1
42	40	3757	c.11270_11274del	p.Leu3757ProfsX56	Frameshift	Extracellular sequence	0	0	2	1
188	40	3789	c.11365_11366del	p.Asn3789TrpfsX25	Frameshift	Extracellular sequence	0	0	2	1

14	40	3793	c.11376dup	p.Thr3793AspfsX22	Frameshift	Extracellular sequence	0	0	2	1
786	40	3793	c.11378del	p.Thr3793SerfsX32	Frameshift	Extracellular sequence	0	0	1	1
676	41	3817	c.11450dup	p.Tyr3817LeufsX142	Frameshift	Extracellular sequence	1	0	1	1
141	41	3843	c.11527del	p.Asp3843ThrfsX101	Frameshift	Extracellular sequence	0	0	1	1
600	42	3851	c.11551del	p.Leu3851TrpfsX93	Frameshift	Extracellular sequence	1	0	1	1
9	42	3888	c.11661dup	p.Ala3888CysfsX72	Frameshift	Cytoplasmic sequence	0	0	3	1
396	42	3895	c.11684del	p.Gly3895AlafsX49	Frameshift	Extracellular sequence	0	0	1	1
572	43	3994	c.11973_11980dup	p.Leu3994ProfsX47	Frameshift	Transmembrane	0	0	1	1
812	43	3995	c.11984_11996del	p.Phe3995SerfsX39	Frameshift	Transmembrane	0	0	1	1
298	44	4014	c.12042del	p.Phe4014LeufsX24	Frameshift	Cytoplasmic sequence	0	0	2	1
322	44	4033	c.12097del	p.Val4033TrpfsX5	Frameshift	Transmembrane	0	0	1	1
36, 159	44	4034	c.12100del	p.Val4034CysfsX4	Frameshift	Transmembrane	0	0	6	2
540	45	4059	c.12175dup	p.Gln4059ProfsX97	Frameshift	Extracellular sequence	1	0	1	1
583	45	4066	c.12197del	p.Pro4066LeufsX131	Frameshift	Extracellular sequence	0	0	1	1
293	45	4073	c.12217_12218del	p.Leu4073ValfsX82	Frameshift	Extracellular sequence	0	0	1	1
77	45	4083	c.12247_12259del	p.Pro4083TrpfsX110	Frameshift	Extracellular sequence	0	0	2	1
94, 501	45	4102	c.12305del	p.Ala4102ValfsX95	Frameshift	Transmembrane	0	0	3	2
522	45	4103	c.12307_12310del	p.Val4103PhefsX93	Frameshift	Transmembrane	0	0	1	1
38, 88	46	4202	c.12605_12632del	p.Arg4202ProfsX146	Frameshift	Cytoplasmic sequence	1	0	2	2

28	46	4224	c.12669_12670del	p.Gln4224ValfsX2	Frameshift	Cytoplasmic sequence	0	0	2	1
167, 382	3	117	c.350T>G	p.Leu117X	Nonsense	Extracellular sequence	0	0	2	2
105, 860	4	135	c.405G>A	p.Trp135X	Nonsense	LRRCT	0	0	4	2
89	5	232	c.696T>A	p.Cys232X	Nonsense	WSC	0	0	1	1
100, 535	5	236	c.706C>T	p.Gln236X	Nonsense	WSC	1	0	3	2
102	5	400	c.1198C>T	p.Arg400X	Nonsense	Extracellular sequence	1	0	1	1
526	6	437	c.1309C>T	p.Gln437X	Nonsense	C-type lectin	0	0	1	1
132	6	439	c.1316G>A	p.Trp439X	Nonsense	C-type lectin	0	0	3	1
243	7	523	c.1568C>A	p.Ser523X	Nonsense	C-type lectin	0	0	2	1
6	9	597	c.1789C>T	p.Gln597X	Nonsense	Extracellular sequence	0	0	2	1
576	10	680	c.2040T>G	p.Tyr680X	Nonsense	Extracellular sequence	0	0	1	1
619	11	718	c.2152C>T	p.Gln718X	Nonsense	Extracellular sequence	1	0	1	1
557	11	901	c.2703G>A	p.Trp901X	Nonsense	PKD 3	0	0	1	1
385	12	987	c.2959C>T	p.Gln987X	Nonsense	PKD4	1	0	1	1
269	13	1023	c.3067C>T	p.Gln1023X	Nonsense	PKD 5	0	0	1	1
2	15	1112	c.3334G>T	p.Glu1112X	Nonsense	PKD 5	0	0	2	1
273	15	1116	c.3346C>T	p.Gln1116X	Nonsense	PKD 5	0	0	1	1
784	15	1172	c.3514C>T	p.Gln1172X	Nonsense	PKD 6	1	0	1	1
631	15	1174	c.3520C>T	p.Gln1174X	Nonsense	PKD 6	0	0	1	1

153	15	1203	c.3607C>T	p.Gln1203X	Nonsense	PKD 6	0	0	2	1
469	15	1436	c.4306C>T	p.Arg1436X	Nonsense	PKD 9	1	1x	1	1
24, 59, 91, 135, 354, 674	15	1483	c.4447C>T	p.Gln1483X	Nonsense	PKD 10	1	0	8	6
403	15	1599	c.4797C>A	p.Tyr1599X	Nonsense	PKD 11	0	novel	2	1
150	15	1621	c.4861C>T	p.Gln1621X	Nonsense	PKD 11	1	0	2	1
66, 127, 439, 552	15	1826	c.5477G>A	p.Trp1826X	Nonsense	PKD 14	0	0	5	4
864	15	1837	c.5510G>A	p.Trp1837X	Nonsense	PKD 14	0	0	1	1
207	15	1874	c.5621G>A	p.Trp1874X	Nonsense	PKD 14	0	0	1	1
543	15	1903	c.5707C>T	p.Gln1903X	Nonsense	PKD 15	1	0	1	1
227	15	2039	c.6115C>T	p.Gln2039X	Nonsense	PKD 16	1	1x	2	1
462, 493	15	2067	c.6199C>T	p.Gln2067X	Nonsense	PKD 17	1	0	2	2
667	15	2164	c.6491C>G	p.Ser2164X	Nonsense	REJ	1	0	1	1
29, 137, 892	15	2184	c.6549dup	p.Glu2184X	Nonsense	REJ	1	0	5	3
156	15	2187	c.6561G>A	p.Trp2187X	Nonsense	REJ	0	0	1	1
81	15	2246	c.6736C>T	p.Gln2246X	Nonsense	REJ	1	0	1	1
454	15	2305	c.6913C>T	p.Gln2305X	Nonsense	REJ	0	0	1	1
35, 43	17	2388	c.7164C>G	p.Tyr2388X	Nonsense	REJ	0	0	3	2
734	18	2405	c.7214G>A	p.Trp2405X	Nonsense	REJ	0	0	1	1
345	18	2430	c.7288C>T	p.Arg2430X	Nonsense	REJ	1	7x	1	1

206	19	2519	c.7555C>T	p.Gln2519X	Nonsense	REJ	1	0	1	1
70, 267	20	2606	c.7816C>T	p.Gln2606X	Nonsense	REJ	1	0	2	2
324, 883	21	2639	c.7915C>T	p.Arg2639X	Nonsense	REJ	1	5x	3	2
903	22	2699	c.8095C>T	p.Gln2699X	Nonsense	REJ	1	0	1	1
629	23	2738	c.8212G>T	p.Glu2738X	Nonsense	REJ	0	0	1	1
452	23	2742	c.8224G>T	p.Glu2742X	Nonsense	REJ	0	0	1	1
797	23	2810	c.8428G>T	p.Glu2810X	Nonsense	REJ	1	3x	1	1
564	23	2824	c.8470C>T	p.Gln2824X	Nonsense	REJ	0	0	1	1
761, 810	27	3183	c.9547C>T	p.Arg3183X	Nonsense	PLAT	1	0	2	2
588	28	3195	c.9585G>A	p.Trp3195X	Nonsense	PLAT	0	0	2	1
388	28	3206	c.9616C>T	p.Gln3206X	Nonsense	PLAT	1	0	1	1
890	30	3350	c.10048A>T	p.Lys3350X	Nonsense	Cytoplasmic sequence	0	0	1	1
145	32	3395	c.10180C>T	p.Gln3394X	Nonsense	Cytoplasmic sequence	1	0	1	1
95	34	3475	c.10420C>T	p.Gln3474X	Nonsense	Cytoplasmic sequence	1	0	2	1
104	34	3478	c.10432G>T	p.Glu3478X	Nonsense	Cytoplasmic sequence	0	0	1	1
45	34	3488	c.10459C>T	p.Gln3487X	Nonsense	Cytoplasmic sequence	1	0	1	1
148	36	3603	c.10806G>A	p.Trp3602X	Nonsense	Transmembrane	0	0	2	1
7	37	3620	c.10855A>T	p.Lys3619X	Nonsense	Cytoplasmic sequence	0	0	2	1
113	38	3702	c.11101C>T	p.Gln3701X	Nonsense	Extracellular sequence	1	0	2	1

567	39	3755	c.11263G>T	p.Glu3755X	Nonsense	Extracellular sequence	0	0	1	1
607	40	3796	c.11388T>A	p.Tyr3796X	Nonsense	Extracellular sequence	0	0	1	1
239	41	3807	c.11420G>A	p.Trp3807X	Nonsense	Extracellular sequence	1	0	5	1
5, 117	41	3808	c.11423G>A	p.Gly3808X	Nonsense	Extracellular sequence	1	0	5	2
75	42	3871	c.11611G>T	p.Glu3871X	Nonsense	Extracellular sequence	1	0	2	1
74	43	3921	c.11763G>A	p.Trp3921X	Nonsense	Cytoplasmic sequence	1	3x	1	1
93	44	4003	c.12007C>T	p.Gln4003X	Nonsense	Cytoplasmic sequence	1	4x	1	1
22	44	4041	c.12121C>T	p.Gln4041X	Nonsense	PKD 3	1	8x	6	1
109	45	4065	c.12195C>A	p.Cys4065X	Nonsense	Extracellular sequence	0	0	1	1
64	45	4126	c.12377dup	p.Tyr4126X	Nonsense	Cytoplasmic sequence	0	0	2	1
210	46	4246	c.12736C>T	p.Gln4246X	Nonsense	Cytoplasmic sequence	0	0	1	1
625	12	951	c.2853+1G>A		Typical splicing	PKD 4	1	0	1	1
549	15	1098	c.3295+1G>T		Typical splicing	PKD 5	0	0	1	1
274	20	2568	c.7703+1G>C		Typical splicing	REJ	0	0	1	1
246, 538, 656, 884	21	2673	c.8017-2_8017-1del		Typical splicing	REJ	1	0	5	4
129	23	2672	c.8017-2A>G		Typical splicing	REJ	1	0	2	1
171	26	3067	c.9201+1G>C		Typical splicing	Transmembrane	1	0	1	1
638	33	3406	c.10217+1G>A		Typical splicing	Cytoplasmic sequence	0	0	1	1
213	32	3476	c.10429-2_10429-1delCA		Typical splicing	Cytoplasmic sequence	0	0	1	1

595	39	3671	c.11014-1del		Typical splicing	Cytoplasmic sequence	0	0	2	1
34	41	3757	c.11270-2A>C		Typical splicing	Extracellular sequence	0	0	3	1
608	44	3903	c.11710-1G>T		Typical splicing	Transmembrane	0	0	1	1
194	44	3904	c.11713-1G>A		Typical splicing	Transmembrane	0	0	1	1
312	45	4000	c.12001-2A>G		Typical splicing	Transmembrane	0	0	1	1
299	4		exon 4	exon 4	Large deletion		0	0	1	1
590	6		exon 6	exon 6	Large deletion		0	0	1	1
316	15		exon 15	exon 15	Large deletion		0	0	1	1
628	15	2219	c.6657_6671del	p.Arg2219_Pro2224del	Large deletion	REJ	1	0	1	1
162	19		exon19 dup	exon19 dup	Large duplication		0	0	2	1
878	21		exon 21	exon 21	Large deletion		0	0	1	1
23	46	3791	c.11373_11390del	p.Gly3791_Ser3797del	Large deletion	Extracellular sequence	0	0	3	1
643	46	4201	c.12601_12628del	p.Gly4201SerfsX147	Large deletion	Cytoplasmic sequence	0	0	1	1

Supplementary Table S6B. PKD1 in-frame insertion/deletions in study families

Family ID	Exon	Codon	cDNA change	Protein change	Domain	PROVEAN score	PKDB	Canada DB	Korean individuals	Korean families	Segregation patients/control
99	36	3565	c.10694_10696del	p.Val3565del	Transmembrane	-7.49	0	0	2	1	

26, 27, 679	10	689	c.2065_2067del	p.Ser689del	Extracellular sequence	-5.37	0	0	6	3	5,0
133	15	2088	c.6258_6263dup	p.Pro2087_Arg2088dup	PKD 17	-5.26	0	0	1	1	1,1
216	15	2217	c.6650_6664del	p.Val2217_Leu2221del	REJ	-16.42	1	0	1	1	
482	15	2251	c.6752_6754del	p.Val2251del	REJ	-7.19	0	0	1	1	
87	20	2613	c.7837_7839del	p.Leu2613del	REJ	-9.98	1	0	3	1	2,0
130	23	2770	c.8308_8310del	p.Asn2770del	REJ	-11.28	0	0	3	1	3,0
124, 208	24	2979	c.8935_8937del	p.Phe2979del	Extracellular sequence	-10.76	1	0	2	2	
37	25	3031	c.9092_9094del	p.Leu3031del	GPS	-14.07	0	0	2	1	2,0

Supplementary Table S6C. PKD1 non-truncating mutations in study families

Family ID	Exon	Codon	cDNA change	Protein change	Domain	SIFT	polyphen	GERP ++	PKDB	Canada DB	Korean individual	Korean family	Segregation patients/control
334	2	75	c.224C>T	p.Ser75Phe	LRR 1	0	1	4.27	1	0	4	1	
355	2	93	c.278T>C	p.Leu93Pro	C-type lectin	0	1	4.27	0	0	1	1	
898	3	120	c.359T>C	p.Ile120Thr	Extracellular sequence	0	0.873	4.26	1	0	1	1	
304	5	381	c.1141G>A	p.Gly381Arg	Extracellular sequence	0.09	0.999	5.05	0	0	1	1	
264	6	419	c.1256G>A	p.Cys419Tyr	C-type lectin	0	1	5.05	0	0	1	1	
186	7	466	c.1396G>A	p.Val466Met	C-type lectin	0.01	1	4.82	1	2x	2	1	

281, 297, 465	7	515	c.1543G>T	p.Gly515Trp	C-type lectin	0	1	4.85	1	0	4	3	
41, 306	7	464	c.1391T>C	p.Leu464Pro	C-type lectin	0.37	0.999	4.82	0	0	3	2	3,0
233	10	693	c.2078G>A	p.Gly693Glu	Extracellular sequence	0.01	1	5.17	0	0	3	1	3,4
519	10	699	c.2095T>C	p.Ser699Pro	Extracellular sequence	0.21	0.943	4.01	0	0	1	1	2,1
342	11	791	c.2371C>T	p.Arg791Trp	PKD 4	0.08	0.999	4.43	0	0	1	1	2,0
616	11	869	c.2605C>T	p.Arg869Cys	PKD 12	0.04	0.992	3.48	0	0	1	1	0,1
248	11	944	c.2830C>T	p.Arg944Cys	PKD 4	0	1	4.96	1	0	1	1	
241	11	845	c.2534T>C	p.Leu845Ser	Extracellular sequence	0	1	5.09	1	0	2	1	
405	12	960	c.2878G>A	p.Gly960Ser	PKD 4	0	1	4.79	1	0	1	1	0,1
444	14	1077	c.3229G>A	p.Val1077Ile	PKD 5	0.23	0.982	5.33	0	0	1	1	
614	14	1055	c.3163T>G	p.Trp1055Gly	PKD 5	0.09	1	5.29	0	0	1	1	
745	15	1587	c.4759C>T	p.Arg1587Cys	PKD 11	0.02	1	4.14	0	0	1	1	
485	15	2085	c.6254C>G	p.Pro2085Arg	PKD 17	0.04	1	5.49	0	0	1	1	
591	15	2215	c.6643C>T	p.Arg2215Trp	REJ	0.01	0.999	5.49	1	1x	1	1	
530	15	2235	c.6704C>T	p.Ser2235Leu	REJ	0	0.999	5.35	0	0	2	1	
624, 848	15	1414	c.4241G>C	p.Trp1414Ser	PKD 9	0.01	1	5.71	0	0	2	2	2,0
332	15	1544	c.4630G>A	p.Val1544Met	PKD 10	0	1	5.36	0	0	2	1	2,0
189	15	1547	c.4640G>A	p.Arg1547His	PKD 10	0.14	1	4.35	0	0	1	1	
219	15	1666	c.4997G>C	p.Trp1666Ser	PKD 12	0	1	5.41	0	0	1	1	

865	15	1832	c.5495G>T	p.Gly1832Val	PKD14	0.01	1	4.67	0	0	1	1	
98	15	2159	c.6475G>T	p.Val2159Leu	REJ	0.74	1	5.49	0	0	1	1	
561	15	1109	c.3327T>A	p.Asn1109Lys	PKD 5	0.01	0.998	-4.05	0	0	1	1	
83	15	1652	c.4955T>C	p.Leu1652Pro	PKD 12	0	1	5.30	0	0	2	1	2,0
261	15	2006	c.6016T>G	p.Trp2006Gly	PKD 16	0	1	5.59	0	0	1	1	
528	15	2132	c.6395T>G	p.Phe2132Cys	PKD 17	0.18	1	4.38	1	0	1	1	
328	18	2445	c.7333A>C	p.Thr2445Pro	REJ	0.13	0.999	4.81	0	0	2	1	
555	18	2434	c.7300C>T	p.Arg2434Trp	REJ	0	1	3.78	1	0	1	1	
348	18	2436	c.7307G>T	p.Gly2436Val	REJ	0.04	1	4.81	0	0	1	1	
569	18	2476	c.7427G>A	p.Cys2476Tyr	REJ	0	1	4.77	0	0	1	1	
513	19	2557	c.7670A>G	p.Asp2557Gly	REJ	0	1	5.14	1	0	1	1	
668	19	2516	c.7546C>T	p.Arg2516Cys	REJ	0.01	1	4.96	1	8x	1	1	
476	19	2497	c.7490G>T	p.Gly2497Val	PKD 16	0	1	4.96	0	0	1	1	
773	19	2526	c.7576T>C	p.Cys2526Arg	REJ	0.24	0.999	4.96	0	0	1	1	
644, 893	23	2863	c.8587A>G	p.Ile2863Val	Extracellular sequence	0.33	0.874	4.89	0	0	2	2	2,0
108, 169, 176, 211, 214, 253, 471, 777, 854	23	2771	c.8311G>A	p.Glu2771Lys	REJ	0.01	1	4.89	0	0	12	9	10,1
231	23	2783	c.8347G>C	p.Ala2783Pro	REJ	0.04	1	4.62	0	0	1	1	
713	24	2963	c.8888T>C	p.Ile2963Thr	Extracellular sequence	0	1	4.55	0	0	2	1	2,0

201, 594	25	3012	c.9034A>C	p.Thr3012Pro	GPS	0.02	1	3.45	0	0	4	2	3,0
677	25	3046	c.9136C>T	p.Arg3046Cys	GPS	0	0.994	4.38	0	0	1	1	
143	25	3043	c.9128G>A	p.Cys3043Tyr	GPS	0	1	4.38	0	0	1	1	
642	25	3014	c.9041T>C	p.Leu3014Pro	GPS	0.12	1	4.55	0	0	1	1	
429	26	3132	c.9395C>T	p.Ser3132Leu	PLAT	0	1	4.51	1	0	1	1	
837	26	3113	c.9338G>A	p.Gly3113Glu	Cytoplasmic sequence	0.02	1	4.51	0	0	1	1	
158, 203	27	3135	c.9404C>T	p.Thr3135Met	PLAT	0	1	4.49	1	0	2	2	
350, 615	28	3194	c.9581C>T	p.Ala3194Val	PLAT	0.01	1	4.84	0	0	2	2	4,0
69	29	3253	c.9758T>C	p.Leu3253Pro	Cytoplasmic sequence	0.01	1	4.69	0	0	2	1	2,0
50	33	3468	c.10402G>A	p.Asp3468Asn	Cytoplasmic sequence	0.01	0.999	4.45	1	0	1	1	
25	36	3591	c.10769C>T	p.Ser3590Phe	Transmembran e	0	1	3.98	0	0	2	1	2,0
192	36	3603	c.10807G>A	p.Glu3603Lys	Cytoplasmic sequence	0.01	1	3.98	1	0	2	1	
585	37	3612	c.10835T>C	p.Leu3612Pro	Cytoplasmic sequence	0.01	1	4.81	0	0	1	1	
236	38	3673	c.11018T>C	p.Leu3673Pro	Extracellular sequence	0.02	0.997	3.46	0	0	2	1	
164	39	3749	c.11246G>A	p.Arg3749Gln	Extracellular sequence	0.07	1	4.04	1	0	1	1	
894	39	3752	c.11255G>A	p.Arg3752Gln	Extracellular sequence	0.08	1	4.04	1	0	1	1	
90	41	3822	c.11465T>A	p.Leu3822Gln	Extracellular sequence	0	1	4.49	0	0	1	1	
212	42	3846	c.11536A>G	p.Ser3846Gly	Extracellular sequence	0.02	0.958	3.88	0	0	1	1	
296	42	3846	c.11538C>G	p.Ser3846Arg	Extracellular sequence	0.01	0.996	3.88	0	0	1	1	

30	42	3853	c.11558T>C	p.Leu3853Pro	Extracellular sequence	0	1	3.97	0	0	4	1	2,0
527	37	3634	c.10901C>A	p.Ala3634Asp	Cytoplasmic sequence	0.57	0.999	3.91	0	0	1	1	
242	4	154	c.461C>T	p.Thr154Met	LRRCT	0	0.547	-3.45	0	0	2	1	
249	15	1768	c.5303C>A	p.Thr1768Asn	PKD 13	0.05	0.984	2.6	0	0	2	1	
496	15	2095	c.6285C>A	p.Asp2095Glu	PKD 17	0.13	0.976	3.51	0	0	1	1	
578	25	2997	c.8991C>G	p.Ser2997Arg	Extracellular sequence	0.07	0.999	3.57	0	0	1	1	
320, 510	37	3650	c.10948G>A	p.Gly3650Ser	Cytoplasmic sequence	0.09	1	3.71	1	0	3	2	
421	15	1706	c.5116G>A	p.Ala1706Thr	PKD 12	0.42	0.506	4.44	0	0	1	1	
466	15	1951	c.5852G>A	p.Arg1951Gln	PKD 15	0.82	0.504	4.24	0	0	1	1	
646	23	2765	c.8294G>A	p.Arg2765His	REJ	0.36	0.543	3.87	0	0	1	1	
289	43	3949	c.11846T>C	p.Leu3949Pro	Transmembrane	0.17	0.999	3.12	0	0	1	1	

Supplementary Table S6D. PKD2 protein-truncating mutations in study families

Family ID	Exon	Codon	cDNA change	Protein change	Predicted Effect	Domain	PKDB	Canada DB	Korean individuals	Korean families
101	1	63	c.187_188insAG	p.Ala63GlufsX55	Frameshift	Cytoplasmic sequence	0	0	1	1
902	1	158	c.471dup	p.Glu158ArgfsX55	Frameshift	Poly-Arg	0	0	1	1
618	1	197	c.588_595del	p.Leu197SerfsX13	Frameshift	Cytoplasmic sequence	0	0	1	1
134	5	381	c.1142del	p.Gly381GlufsX71	Frameshift	Extracellular sequence	0	0	2	1

329	7	569	c.1704dup	p.Val569CysfsX4	Frameshift	Transmembrane	0	0	4	1
72	11	710	c.2127dup	p.Lys710X	Frameshift	EF-hand domain	0	0	1	1
152	11	714	c.2142del	p.Lys714AsnfsX2	Frameshift	EF-hand domain	0	0	1	1
97	11	720	c.2159dup	p.Asn720LysfsX5	Frameshift	EF-hand domain	0	0	2	1
346	11	722	c.2164del	p.Val722TrpfsX15	Frameshift	EF-hand domain	0	0	1	1
220	11	738	c.2213_2214del	p.Phe738X	Frameshift	EF-hand domain	0	0	1	1
366	11	747	c.2240del	p.Lys747ArgfsX23	Frameshift	EF-hand domain	0	0	1	1
80	1	80	c.239C>A	p.Ser80X	Nonsense	Cytoplasmic sequence	0	0	1	1
596, 641	1	87	c.260G>A	p.Trp87X	Nonsense	Poly-Glu	0	0	2	2
32, 154, 437	1	183	c.547C>T	p.Gln183X	Nonsense	Cytoplasmic sequence	0	0	5	3
474	2	201	c.603G>A	p.Trp201X	Nonsense	Transmembrane	1	0	1	1
727	2	223	c.667G>T	p.Glu223X	Nonsense	Cytoplasmic sequence	0	0	1	1
10, 654	3	266	c.796G>T	p.Glu266X	Nonsense	Extracellular sequence	1	0	3	2
58, 222, 565	4	306	c.916C>T	p.Arg306X	Nonsense	Extracellular sequence	1	0	3	3
389	4	320	c.958C>T	p.Arg320X	Nonsense	Polycystin motif	1	7x	1	1
142, 237	4	361	c.1081C>T	p.Arg361X	Nonsense	Extracellular sequence	1	0	4	2
40	5	417	c.1249C>T	p.Arg417X	Nonsense	Extracellular sequence	1	10x	2	1
31, 163, 836	6	455	c.1365G>A	p.Trp455X	Nonsense	Extracellular sequence	1	0	4	3
651, 660	6	464	c.1390C>T	p.Arg464X	Nonsense	Extracellular sequence	1	0	3	2

270	9	654	c.1960C>T	p.Arg654X	Nonsense	Extracellular sequence	1	5x	1	1
275	10	684	c.2052C>A	p.Tyr684X	Nonsense	Cytoplasmic sequence	0	0	2	1
106, 198, 479, 626, 838	10	701	c.2102C>G	p.Ser701X	Nonsense	Cytoplasmic sequence	0	0	5	5
18, 205, 546, 573	11	742	c.2224C>T	p.Arg742X	Nonsense	EF-hand domain	1	0	6	4
12	12	776	c.2326C>T	p.Gln776X	Nonsense	EF-hand domain	0	0	2	1
123, 155, 558, 636	13	803	c.2407C>T	p.Arg803X	Nonsense	Linker	1	6x	10	4
140	14	874	c.2620A>T	p.Lys874X	Nonsense	Coiled coil	0	0	1	1
86	1	198	c.595+1G>C		Typical splicing	Cytoplasmic sequence	0	0	1	1
372, 633	4	365	c.1094+1G>T		Typical splicing	Extracellular sequence	0	0	2	2
44	5	365	c.1095-2A>G		Typical splicing	Extracellular sequence	1	0	4	1
460	5	365	c.1095-2A>T		Typical splicing	Extracellular sequence	0	0	1	1
319	6	516	c.1548+1G>A		Typical splicing	Transmembrane	0	0	1	1
401	8	572	c.1717-2A>C		Typical splicing	Cytoplasmic sequence	0	0	1	1
3	11	747	c.2240+1G>T		Typical splicing	EF-hand domain	1	0	2	1
349	1		exon 1	exon 1	Large deletion		0	0	1	1
11	2		Intron2-exon5	p.M1fsX	Large deletion	Cytoplasmic sequence	0	0	8	1
574, 609	3		Exon 3-5	Exon 3-5	Large deletion		0	0	2	2
897	8		exon 8	exon 8	Large deletion		0	0	1	1

Supplementary Table S6E. PKD2 non-truncating mutations in study families

Family ID	Exon	Codon	cDNA change	Protein change	Domain	SIFT	Polyphen	GERP ++	PKDB	CanadaDB	Korean individuals	Korean families	Segregation patients/control
435	1	179	c.536C>A	p.Pro179His	Cytoplasmic sequence	0.01	0.838	3.98	0	0	1	1	
13	4	290	c.869G>T	p.Gly290Val	Extracellular sequence	0	0.99	5.07	0	0	4	1	2,0
335, 340	4	322	c.965G>A	p.Arg322Gln	Polycystin motif	0	0.999	5.62	1	0	5	2	
82, 899	4	325	c.974G>A	p.Arg325Gln	Polycystin motif	0.01	0.988	-3.84	1	3x	4	2	
240	5	389	c.1166C>T	p.Ala389Val	Extracellular sequence	0	0.907	4.9	0	0	2	1	3,1
394	5	374	c.1121T>G	p.Leu374Trp	Extracellular sequence	0	0.892	4.9	0	0	1	1	
122, 292	5	381	c.1141G>A	p.Gly381Arg	Extracellular sequence	0	0.966	4.9	0	0	3	2	3,1
282, 472	14	890	c.2668G>A	p.Glu890Lys	Coiled coil	0.21	1	5.01	0	0	2	2	2,1

Supplementary Table S7. Definition of Grades

Grade of Mutations	
Definitely pathogenic (DP)	• Stop-gain single-nucleotide variants (SNVs)<br>• Frameshift indels<br>• In-frameshift indels with ≥5 amino acids<br>• Typical splice site mutations<br>• Either newly discovered from our samples or previously registered in the Mayo DB or TGESP(1) data
Highly likely pathogenic (HLP)	• Nonsynonymous SNVs or in-frameshift indels of <5 amino acids that were established as HLP in the Mayo DB or TGESP DB<br>• Novel LP variants segregated in family(s)
Likely pathogenic (LP)	• Nonsynonymous SNVs that were listed as LP in Mayo DB,<br>• Possibly causal nonsynonymous SNVs that satisfied 2 of the following 3 conditions<br>➢ Being damaging, as predicted by a SIFT score ≤0.05<br>➢ Being damaging, as predicted by Polyphen-2 ([HumDiv] ≥0.453 or [HVAR] ≥0.447)<br>➢ Having a genomic evolutionary rate profiling [GERP] ++ score ≥2<br>• Possibly causal in-frameshift indels of <5 amino acids that satisfied Protein Variation Effect Analyzer PROVEAN] score of ≤2.5
**No mutations (NM)**	
Likely neutral (LN)	• Previously registered in the Mayo DB or TGESP DB<br>• Newly found in normal controls
Indeterminate (I)	• Nonsynonymous SNV previously registered in the Mayo DB or TGESP DB<br>• Nonsynonymous SNV or in-frameshift indels of <5 amino acids with obscure pathogenicity in SIFT, Polyphen-2 [HumDiv or HVAR], [GERP] ++, or [PROVEAN]
No variant (NV)	• No possible pathogenic variants

Supplementary Table S8. MLPA validation using Long Range PCR sequencing

1. Long Range PCR information

Exon	Forward primer	Reverse primer	Tm ('C)	Product size (bp)
**2-7**	5'-CCCCGAGTAGCTGGAACTACAGTTACACACT-3'	5'-CGTCCTGCTGTGCCAGAGGCG-3'	70	4041
**13-15**	5'-TGGAGGGAGGGACGCCAATC-3'	5'-GTCAACGTGGGCCTCCAAGT-3'	65	4391
**15-21**	5'-ATCCCTGGGGGTCCTACCATCTCTTA-3'	5'-ACACAGGACAGAACGGCTGAGGCTA-3'	68	5253

2. Sequencing primers information

Exon	primer name	Sequence	Genomic position (of 5' first bp; hg19)	Distance to exon-intron junction
2-3	PKD1_2-3-F	5'-GGGATGCTGGCAATGTGTGGGAT-3'	2,169,468	- 89 exon 2
	PKD1_2-3-R	5'-GGACCAACTGGGAGGGCAGAA-3'	2,169,038	77 exon 2
4	PKD1_4-F	5'-GGCGGTGCTGTCAGGGTG-3'	2,168,948	- 102 exon 4
	PKD1_4-R	5'-CCAGAGAGGCCTTCCTGAGC-3'	2,168,582	95 exon 4
5(A)	PKD1_5A-F	5'-GAACAGCATGGGAGCCTGTGAGT-3'	2,168,561	- 95 exon 5
	PKD1_5A-R	5'-AGCCGGCCCAGCGGCATC-3'	2,168,033	Within an exon 5
5(B)	PKD1_5B-F	5'-GCCTGTCCCTCTGCTCCG-3'	2,168,262	Within an exon 5
	PKD1_5B-R	5'-GTGTCAACGGTCAGTGTGGGC-3'	2,167,696	96 exon 5
6	PKD1_6-F	5'-GTGTCTGCTGCCCACTCCC-3'	2,167,787	- 124 exon 6
	PKD1_6-RF	5'-CTCCTTCCTCCTGAGACTCCC-3'	2,167,397	93 exon 6
15(A)	PKD1_15A-F	5'-TTCTGCCGAGCGGGTGGG-3'	2,161,938	- 64 exon 15
	PKD1_15A-R	5'-CATGTCGAAGGTCCACGTGATGT-3'	2,161,427	Within an exon 15
15(B)	PKD1_15B-F	5'-GACATGAGCCTGGCCGTGG-3'	2,161,516	Within an exon 15
	PKD1_15B-R	5'-CCACCTCTGGCTCCACGCA-3'	2,161,027	Within an exon 15
15(C)	PKD1_15C-F	5'-CACGCGGAGCGGCACGTT-3'	2,161,121	Within an exon 15
	PKD1_15C-R	5'-GGTGACCTCCGGACCCTC-3'	2,160,626	Within an exon 15
15(D)	PKD1_15D-F	5'-TCTGCTGTGGGCCGTGGG-3'	2,160,706	Within an exon 15
	PKD1_15D-R	5'-CTGTACCGTGTGGTTGGTGGG-3'	2,160,215	Within an exon 15
15(E)	PKD1_15E-F	5'-ACAGCATCTTCGTCTATGTCCTG-3'	2,160,303	Within an exon 15
	PKD1_15E-R	5'-GGTTCCCTGCCGTCATGGTG-3'	2,159,812	Within an exon 15

15(F)	PKD1_15F-F	5'-GGGCTGAGCTGGGAGACCT-3'	2,159,899	Within an exon 15
	PKD1_15F-R	5'-GACAGCTGAGCCGGCAGC-3'	2,159,417	Within an exon 15
15(G)	PKD1_15G-F	5'-CTGTGGGCCAGCAGCAAGGT-3'	2,159,494	Within an exon 15
	PKD1_15G-R	5'-CGTGCGGTTCTCACTGCCCA-3'	2,159,012	Within an exon 15
15(H)	PKD1_15H-F	5'-GACGTCACCTACACGCCCG-3'	2,159,095	Within an exon 15
	PKD1_15H-R	5'-CCTCCCAGCGGTACTCAGTCT-3'	2,158,603	Within an exon 15
15(I)	PKD1_15I-F	5'-GATGCGGCGATCACAGCGCA-3'	2,158,688	Within an exon 15
	PKD1_15I-R	5'-GGCCAGCCCTGGTGGCAA-3'	2,158,164	Within an exon 15
21	PKD1_21-F	5'-AGTCGTGGGCATCTGCTGGC-3'	2,155,550	- 75 exon 21
	PKD1_21-R	5'-CAAGCTGCCCGTCTGCCCT-3'	2,155,240	83 exon 21

Reference. Ying-Cai Tan et al., J Mol Diagn. 2012 Jul;14(4):305-13. doi: 10.1016/j.jmoldx.2012.02.007

Supplementary Figure S1

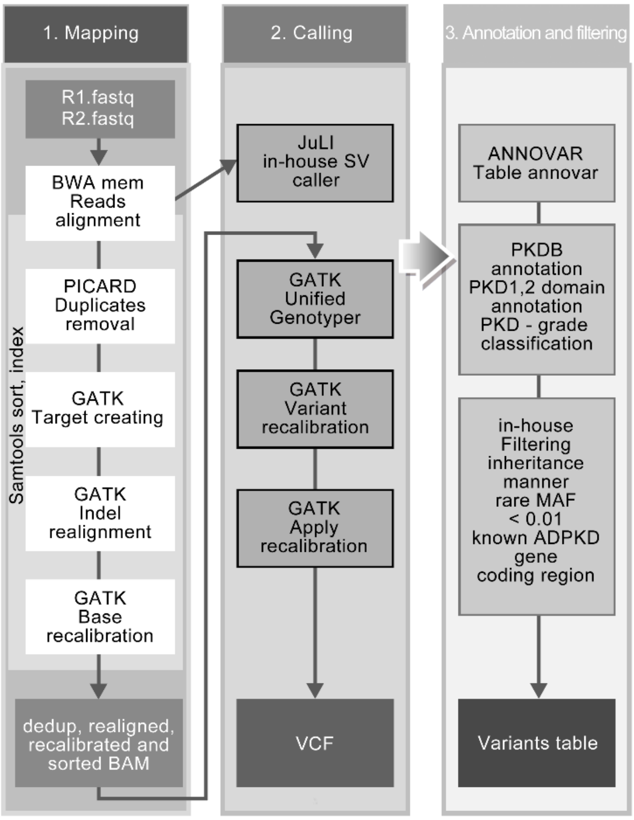

Sequencing reads were first aligned with the human genome reference sequence (hg19) using BWA version 0.7.5a with the MEM algorithm (default options). To minimize false-positive and false-negative calls, we only used uniquely mapped reads that were properly paired to avoid mapping reads that aligned to both target regions and pseudogenes. In addition, only selected reads with a high mapping quality (>30) were used. SAMTOOLS version 1.2 and Picard version 1.127 (http://picard.sourceforge.net) were used to process SAM/BAM files to duplicate the marking. Specifically, local realignment to reduce any misalignment in the duplicated regions was performed.

We used RealignerTargetCreator and IndelRealigner from GATK version 3.3-1 with known single-nucleotide polymorphisms (SNPs) and indels from dbSNP142, Mills and 1000G gold-standard indels at hg19 sites, and 1000G phase 1 indels at hg19 sites. Known SNPs and indels were also used to perform a base calibration. For calling variants, Unified Genotyper was

used, and the called variants were recalibrated by GATK based on dbSNP142, Mills indels, HapMap, and Omni. ANNOVAR was used to annotate the variants. The depth of coverage approach was applied to detect any large rearrangement or copy number variations. The CalculateHsMetrics module in PICARD was used to calculate the statistics of each parameter of the samples and depth of each exon, and the copy number ratio was characterized by the normalized depth of the exons from all the patients

Supplementary Figure S2

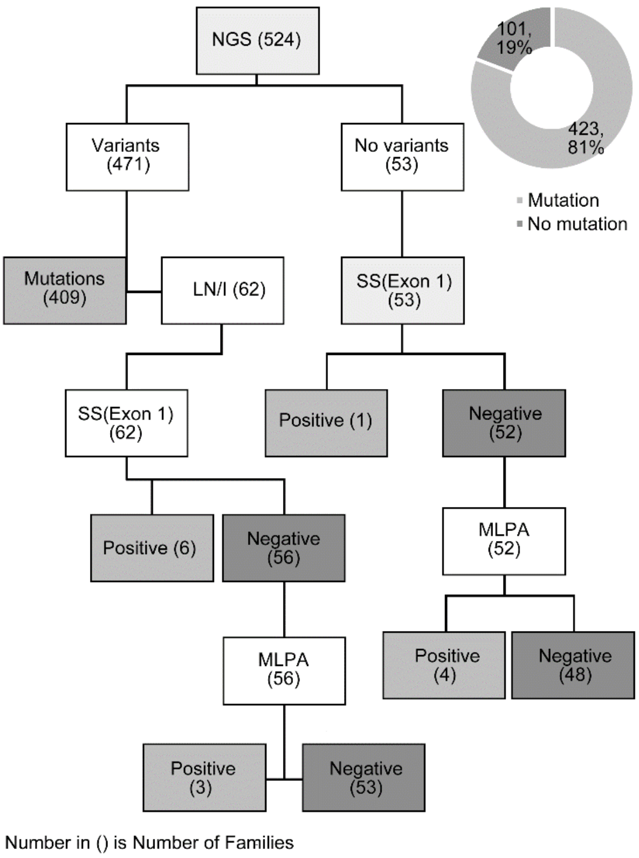

Of the entire sample of 524 families, variants were found by targeted exome sequencing (TES) in 471 families (89.9%). Among them, mutations were found in 409 families. In 62 families with likely neutral (LN) or indeterminate (I) variants and 53 families with no variants (NV), subsequent *PKD1* exon 1 Sanger sequencing (SS) and/or multiple ligation probe assay (MLPA) revealed 10 additional mutations. An overall mutation detection rate was 80.7% (423/524 families) by TES, *PKD1* exon 1 Sanger sequencing, and MLPA.

Supplementary Figure S3

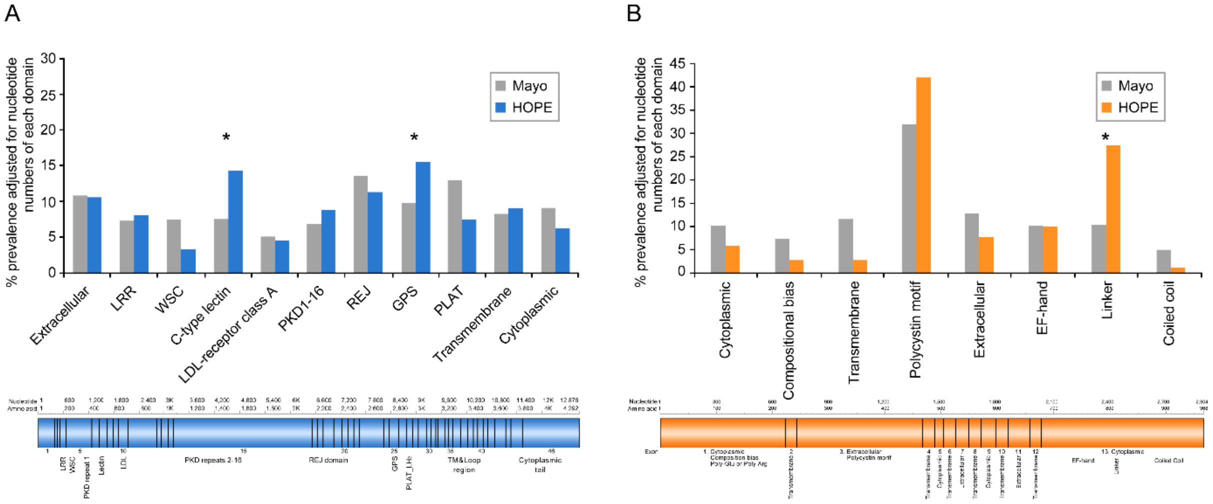

Mutations were found more frequently in the C-type lectin and G protein-coupled receptor proteolytic site (GPS) domains of polycystin-1 in our cohort compared to those reported from the ADPKD mutation database (PKDB). Likewise, mutations were detected more frequently in the linker domain of polycystin-2 in our patients compared to those reported from PKDB. The standardized residual of the chi-square test (cut-off of significance, 2.58) was 2.8 in the C-type lectin of polycystin-1 and the linker domain of polycystin-2, and 2.6 in the GPS domain of polycystin-1.

Supplementary Figure S4

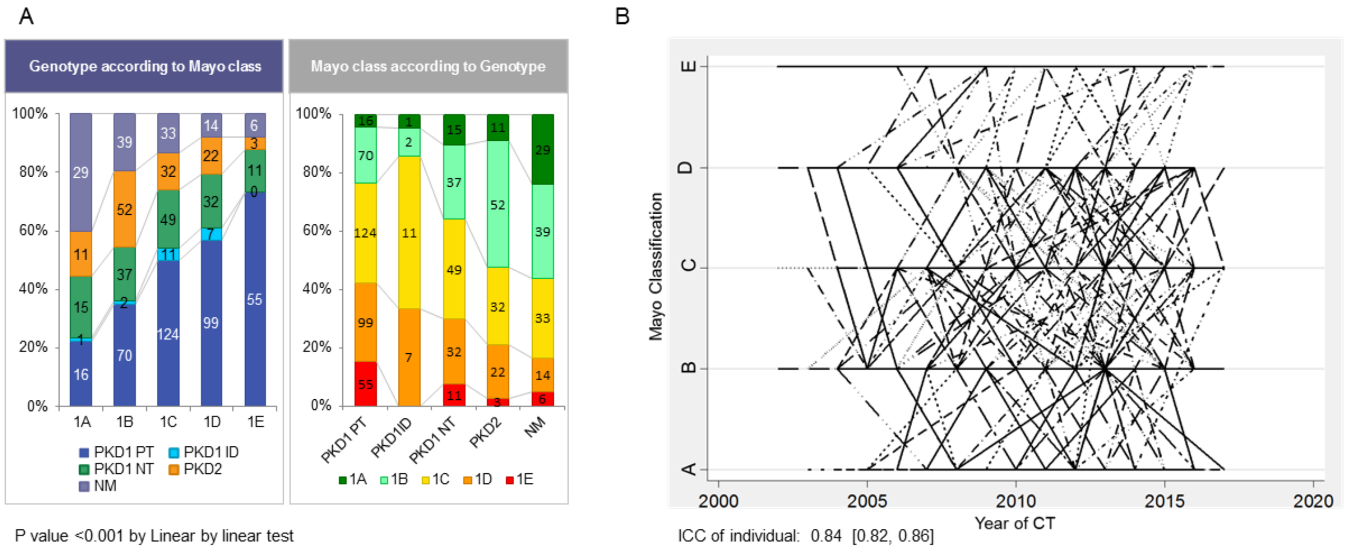

A. As the Mayo imaging classification changed from 1A to 1E, the proportion of *PKD1*-protein truncating (*PKD1*-PT) genotype increased. Conversely, as the genotype changed from *PKD2* to *PKD1*-PT, the proportion of Mayo imaging classification 1C-E increased (P <0.001 by linear by linear test). B. This figure shows the longitudinal distribution of Mayo class of individuals. For the median follow up duration, the ICC of the intraclass correlation of individuals was 0.84 [0.82, 0.86] showing that Mayo imaging classification of each subject was mostly retained over time.

Abbreviations. PKD1-PT, PKD1 protein truncating; PKD1 ID, PKD1 small in-frameshift indels; PKD1-NT, PKD1 non-truncating; NM, no mutations.

Supplementary Figure S5

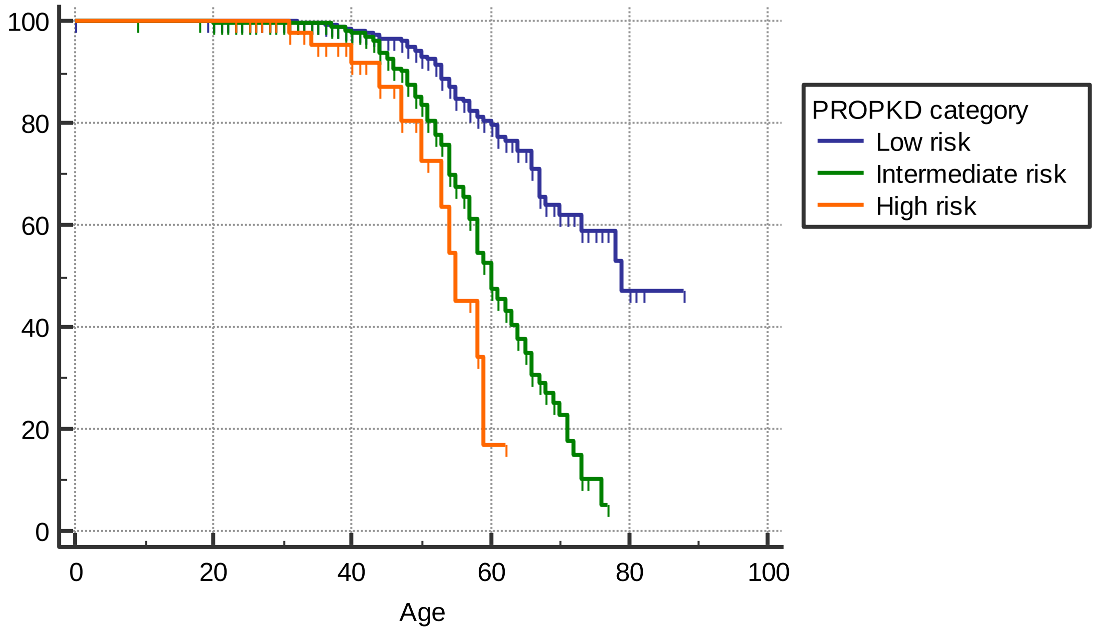

Number at risk

	0	20	40	60	80	100
Group: Low risk	320	318	260	103	5	0
Group: Intermediate risk	316	310	234	44	0	0
Group: High risk	51	51	24	1	0	0

Supplementary Figure S6

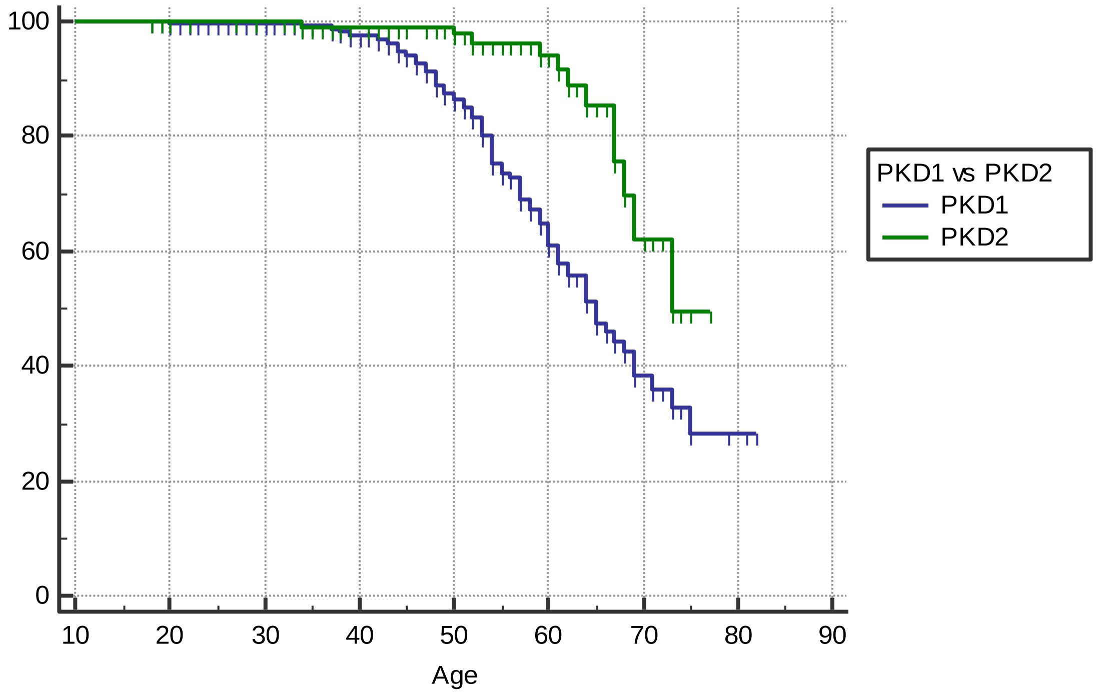

Number at risk

Group: PKD1									
	511	506	467	373	210	72	16	2	0
Group: PKD2									
	119	116	112	96	71	37	7	0	0

The ESRD incidence was 21.5% (110/511) in PKD1 and 10.1% (12/119) in PKD2. The PKD1 genotype showed an earlier onset of ESRD than PKD2 genotype (64.9 vs. 72.9 years old, P<0.001).

Supplementary Figure S7

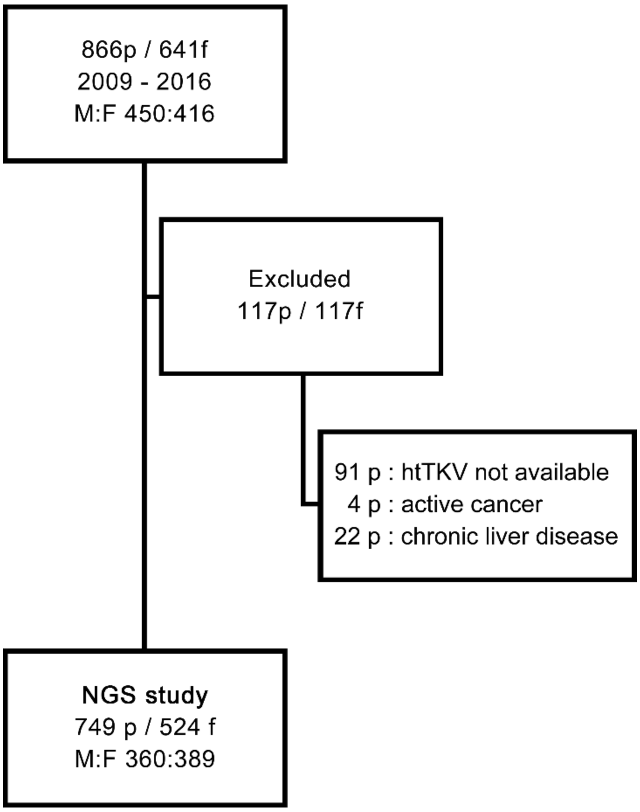

A total of 866 subjects from 641 unrelated families registered in the HOPE-PKD cohort between 2009 and 2016 were screened. Among the screened patients, 117 patients were excluded from the analysis including those who had active cancer (n=4) or chronic liver disease (n=22) and those who had no kidney or liver volume data available (n=91). A total of 749 subjects from 524 unrelated families were included in the analysis.

Supplementary Figure S8

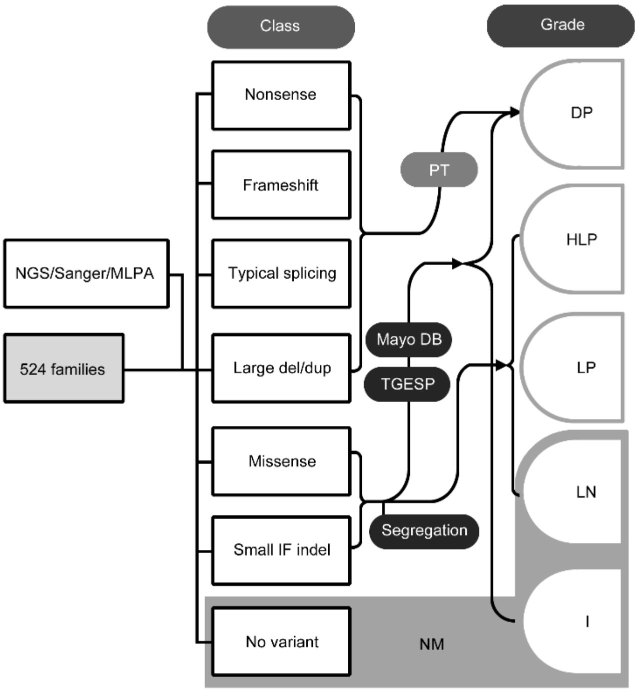

The detected variants were classified into seven categories of nonsense, frameshift, typical splicing, large deletion, missense, small in-frame indel and no variant. Seven classes of variants were further divided into five grades according to their pathogenicity: definitively pathogenic (DP), highly likely pathogenic (HLP), likely pathogenic (LP), likely neutral (LN), and indeterminate (I). The DP variants were defined as all the variants that result in protein truncation, including nonsense, frameshift, typical splicing, and large deletion variants or those previously established as DP in the ADPKD mutation database (PKDB) or previous articles. The HLP variants were defined as missense variants or small in-frame indels that were previously established as HLP in PKDB or previous articles. The LP variants were defined as missense variants or small in-frame indels that were previously established as LP in PKDB or previous articles or variants that were predicted to be damaging using in-silico analysis and segregated within a family. Variants were thought to be damaging if the results of in-silico analysis satisfied two of the following conditions: 1) damaging as predicted by a

SIFT score ≤0.05, 2) damaging as predicted by Polyphen-2 (HumDiv), and 3) GERP ++ score ≥4.

Supplementary Figure S9

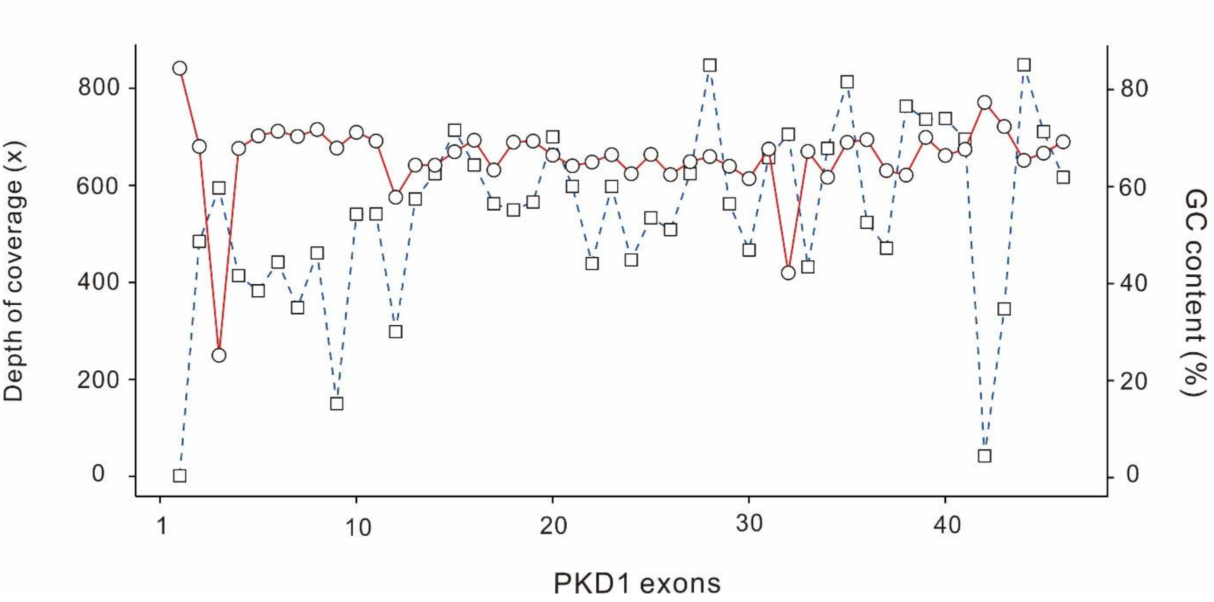

The problem of NGS was low read depth or coverage of exon 1 of PKD1 gene. This resulted from high GC contents of exon 1 compared to other exons.

Supplementary Figure S10

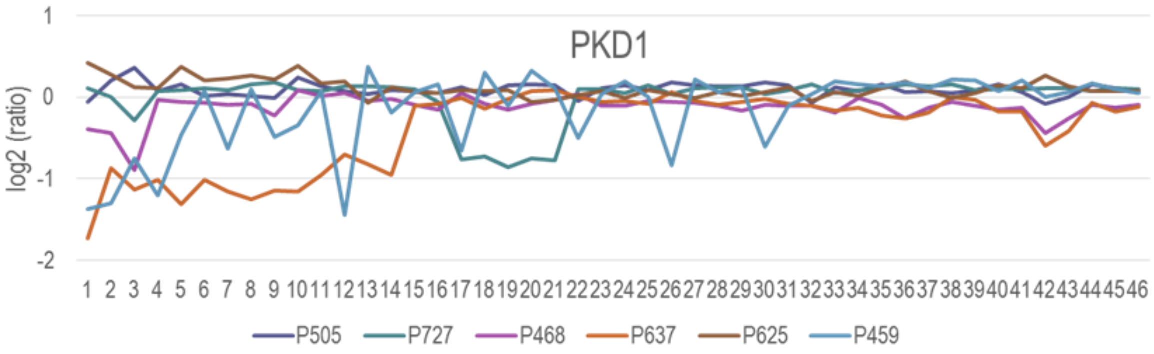

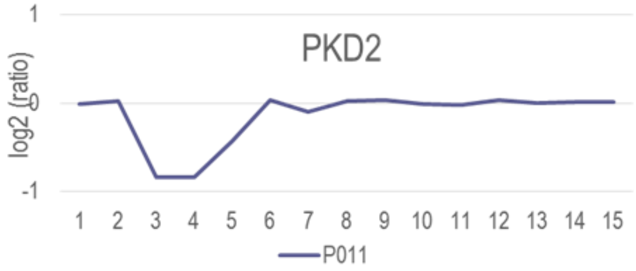

	MLPA	NGS
P459	exon 4	exon 1-4
P468	exon 15	Not detected
P505	exon 15	Not detected
P625	exon 3-5	Not detected
P637	exon 6	exon 1-14
P727	exon 21	exon 17-21
P011	exon 3-5	exon 3-5
